# Optimizing carbon dioxide enrichment with environmental management to improve photosynthesis, water use efficiency and yield in cucumbers: a meta-analysis study

**DOI:** 10.3389/fpls.2026.1826536

**Published:** 2026-05-28

**Authors:** Xin Liu, Xinying Liu, Yaliang Xu, Zheng Wang, Qiying Sun, Sujun Liu, Binbin Liu, Qingming Li

**Affiliations:** 1 Institute of Urban Agriculture, Chinese Academy of Agricultural Sciences, Chengdu, China; 2 Key Laboratory of Plant Factory Speed Breeding, Ministry of Agriculture and Rural Affairs, Chengdu, China; 3 National Chengdu Agricultural Science and Technology Center, Chengdu, China; 4 College of Food and Biological Engineering, Chengdu University, Chengdu, China

**Keywords:** carbon dioxide enrichment, cucumber, meta-analysis, photosynthesis, water use efficiency, yield

## Abstract

Despite the ongoing increase in atmospheric carbon dioxide concentrations, these levels remain well below the optimal threshold for cucumber growth and development. To address the challenges posed by climate change to agricultural production, carbon dioxide enrichment (eCO_2_) has been widely used to meet the global demand for vegetables. However, the effects of eCO_2_ and environmental factors on cucumbers remain unclear. In this study, we conducted a meta-analysis of 73 studies to analyse the responses of cucumber photosynthesis, biomass accumulation and yield to eCO_2_. Our findings revealed that eCO_2_ significantly increased the net photosynthetic rate by 56.31%, water-use efficiency by 121.11%, biomass by 27.75% and yield by 21.98%. The optimal eCO_2_ concentration for cucumber production was found to be 800–1200 ppm. Combined application with high light intensity, a suitable temperature, humidity and fertiliser was found to have a synergistic effect. Our findings support the regulation of greenhouse environments for cucumber cultivation in the context of climate change, promoting the green and sustainable development of the cucumber industry.

## 1 Introduction

The global climate system is undergoing unprecedented and profound changes, and ongoing climate change has become a core challenge constraining sustainable agricultural production. According to the Sixth Assessment Report released by the Intergovernmental Panel on Climate Change (IPCC) (IPCC, 2023) and the research findings (Horton et al., 2021), the sharp rise in atmospheric carbon dioxide concentrations is one of the most significant features of current climate change. CO_2_ serves as the core raw material for plant photosynthesis, playing an irreplaceable role in carbohydrate synthesis, energy conversion, and material metabolism. Despite atmospheric carbon dioxide concentrations having historically exceeded 400 ppm, research indicates that this concentration level is far from optimal for the growth and development of most vegetable crops (Syed and Hachem, 2019). In scenarios involving controlled environment agriculture, shortages of carbon dioxide are particularly evident. Due to the enclosed nature of facility environments and limitations in air circulation, the carbon dioxide circulation system within facilities differs significantly from that of the external atmosphere. This sustained low-concentration environment leaves facility vegetables in a state of carbon starvation, severely limiting the accumulation of photosynthetic products and plant growth and development, thereby becoming a key bottleneck constraining the quality and efficiency of facility agriculture (Zhang et al., 2014). To address the challenges posed by climate change to agricultural production whilst meeting the growing global demand for vegetable supply, carbon dioxide fertilization technology has been gradually applied in horticultural facilities since the early 20th century. Following a period of over a century characterized by technological iteration and practical exploration, this technology has evolved into a significant means of enhancing the yield and quality of greenhouse vegetables (Dong et al., 2018; Hao et al., 2020).

In the global vegetable industry landscape, cucumbers have always been of significance due to their diverse consumption scenarios and massive market demand. The global area under cucumber cultivation has been steadily increasing, making it one of the core drivers for ensuring stable vegetable supply and promoting agricultural economic development (Zhao et al., 2019). However, in the current context of increasingly widespread protected cultivation, cucumber production faces numerous challenges posed by environmental constraints. While controlled environment agriculture provides relatively controllable conditions such as temperature, humidity, and light for cucumber growth, the enclosed nature of such spaces leads to imbalances in carbon dioxide circulation. As a typical C3 plant, the Calvin cycle in cucumber leaves relies on carbon dioxide as a carbon source and is highly sensitive to changes in carbon dioxide concentration (Chen et al., 2022). In facility cultivation scenarios, after sunrise, as photosynthesis in plants rapidly begins, carbon dioxide concentrations inside the facility drop sharply within a short period of time (Zhang et al., 2020). At this point, the carbon dioxide concentration within the facility environment is far below the atmospheric background value, causing the net photosynthetic rate of cucumber leaves to decrease by approximately 35-40% (Chen et al., 2019). Prolonged exposure to this low-carbon dioxide environment has been demonstrated to significantly reduce the carbon assimilation capacity of cucumber plants, directly leading to a decrease in cucumber yield of approximately 20-30%, while the nutrient content of the fruit is also negatively impacted to varying degrees (Kläring et al., 2007).

It is generally accepted that eCO_2_ have a positive impact on photosynthetic rate and yield (Li et al., 2025). eCO_2_ of cucumbers at concentrations of 400–1600 ppm can increase photosynthetic rate and yield of cucumbers to varying degrees (Liu et al., 2018; Abdeldaym et al., 2024). The increase in yield was attributed to the increase in net photosynthetic rate and water utilization, which promoted leaf growth, increased plant yield, and plant tolerance to stressful environments such as heat and drought (Huang and Xu, 2015). However, the effects of eCO_2_ do not exist in isolation, and there are complex interactions between its effects and environmental factors such as light intensity, humidity, and temperature. Other environmental factors such as light and humility affect the effect of eCO_2_ on cucumber, while, at the same time, eCO_2_ interacts with other environmental effects on cucumber (Long et al., 2006). Therefore, the synergistic and antagonistic relationships between eCO_2_ and environmental factors make the response of cucumber to CO_2_ enrichment highly complex and dynamic.

A significant number of studies have focused on food crops such as wheat and soybeans (Ziska and Bunce, 2007; Wang et al., 2013). However, limited number of studies have conducted comprehensive analyses of the effects of eCO_2_ on vegetables, and there is an even greater lack of specific analyses on cucumbers alone. Despite the plethora of reports on the effects of eCO_2_ on cucumber growth, systematic studies on the coupled effects of different eCO_2_ concentrations with environmental factors remain insufficient. In particular, the regulatory mechanisms of cucumber physiological metabolism under the interactive effects of eCO_2_ with factors such as light, water, and temperature remain unclear. Consequently, a thorough literature review is imperative to elucidate the effects of eCO_2_ on cucumber growth, photosynthesis, and yield, as well as its interactive effects with the environment. This not only contributes to refining the theoretical framework for environmental regulation in protected cucumber cultivation but also provides a theoretical basis and practical guidance for the development of precise and intelligent CO_2_ fertilization techniques. This holds significant scientific and practical value for promoting the green and sustainable development of the cucumber industry.

## 2 Materials and methods

### 2.1 Data collection

**Figure 1:**
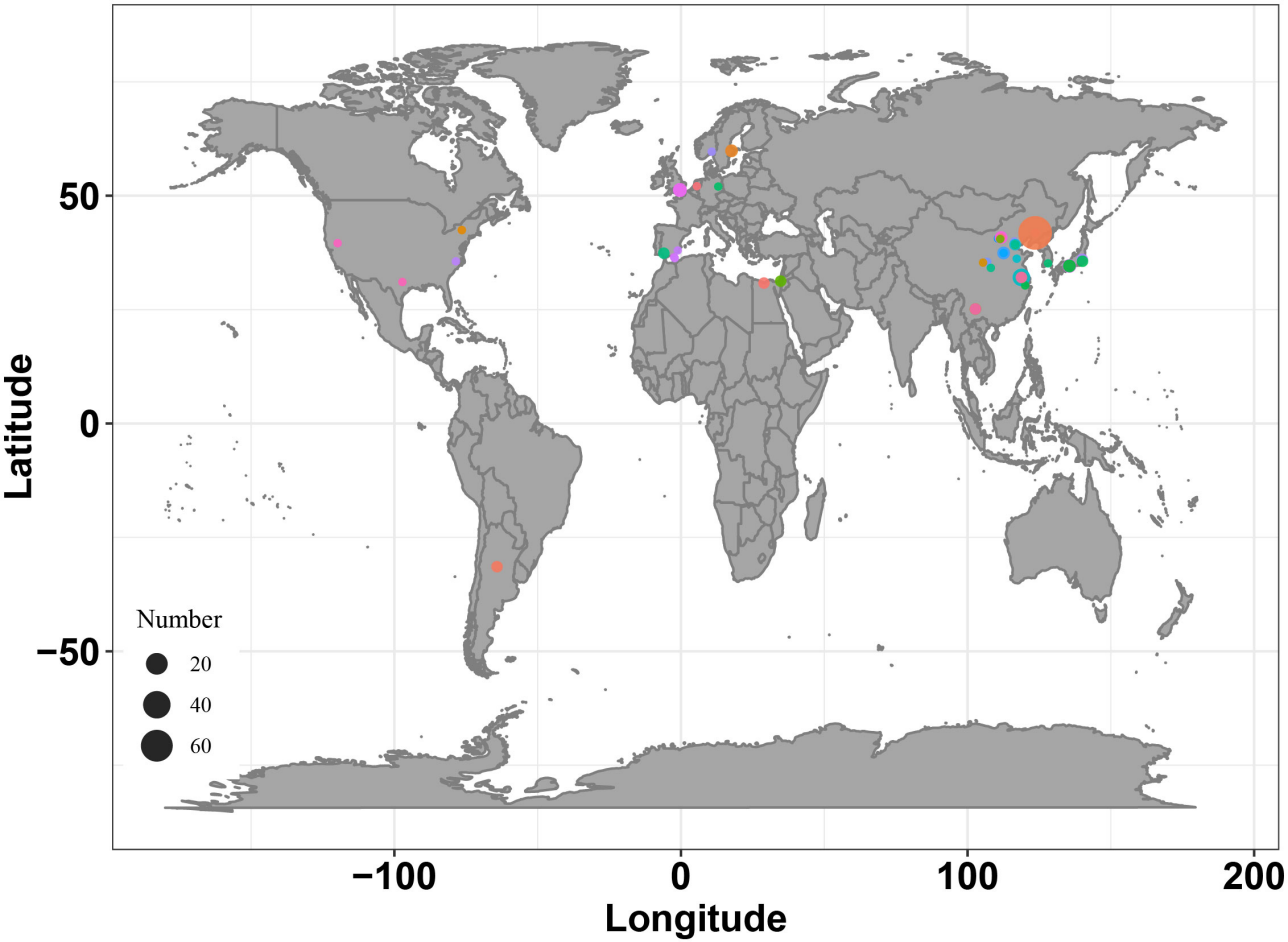
Spatial distribution of published papers, with locations represented by points. The size of the points represents the size of the sample size for different studies.

We searched for peer-reviewed journal articles using the “China National Knowledge Infrastructure”, “Web of Science”, “Science Direct”, and “Google Scholar” (as of January 2025), with the following keyword combinations: (elevated carbon dioxide OR eCO_2_ OR CO_2_ enrichment OR carbon dioxide enrichment) and (cucumber). Articles must be selected as eligible studies based on the following criteria: (i) Research paper. (ii) The study included both control and CO_2_ enrichment treatments. (iii) At least one variable was recorded including plant height, Stem diameter, leaf area, biomass, photosynthesis indicators and yield. (iv) Each treatment was replicated at least three times. After a second screening by reading the titles and abstracts of the literature and excluding those that did not meet the selection criteria, the retained literature was subjected to a more rigorous screening. The entire flowchart of the review program is shown in **Supplementary Figure 1**. Publications and datasets can be found in **Supplementary Table 1**. Finally, 73 articles from around the world met our criteria and were used in our meta-analysis (**Figure 1**).

We further extracted light intensity, temperature, humidity, and concentrations of nitrogen (N), phosphorus (P) and potassium (K) fertilizers in the nutrient solution to assess the effects of CO_2_ enrichment on cucumber growth, photosynthesis, and yield. All target variables (mean, standard error, sample size) were displayed graphically or numerically and data presented graphically were extracted using WebPlotDigitizer (Burda et al., 2017). In instances where the paper did not mention the standard deviation (SD) or standard error (SE) of the variable, the metagear package was utilized to estimate the SD (Lajeunesse, 2016)).

### 2.2 Meta-analysis

Effect sizes were calculated using the natural logarithm of response ratios (lnRR) according to the meta-analysis method (Hedges et al., 1999). The calculation formula is as follows Equation 1:

(1) $$\begin{array}{c} lnRR=ln(\frac{X_{t}}{X_{c}})=ln(X_{t})-ln(X_{c}) \end{array}$$

*X_t_* and *X_c_* are the mean values of treatment and control, respectively. The variance (V_lnRR_) for each effect size was calculated as follows Equation 2:

(2) $$\begin{array}{c} V_{lnRR}=\frac{S_{t}^{2}}{n_{t}{\bar{X}}_{t}^{2}}+\frac{S_{c}^{2}}{n_{c}{\bar{X}}_{c}^{2}} \end{array}$$

Where *n_t_* and *n_c_* are the sample sizes of the treatment and control groups, respectively, and *s_t_* and scare the standard deviations of the treatment and control groups, respectively.

Weighted averages are used to produce maximum accuracy and can eliminate as much variation as possible. The weighted average response ratio (lnRR_+_) and weights (W_i_) were calculated from Equations 3, 4, respectively.

(3) $$\begin{array}{c} lnRR_{+}=\sum_{i=1}^{k}w_{i}\times lnRR/\sum_{i=1}^{k}w_{i} \end{array}$$

(4) $$\begin{array}{c} w_{i}=\frac{1}{V_{lnRR}} \end{array}$$

Here i and k denote the number of comparisons and cumulative studies, and *w_i_* denotes the weight of independent studies. Weighted effect ratio variance (Var (lnRR_+_)) and 95% confidence intervals (95% CI) were calculated using Equations 5, 6.

(5) $$\begin{array}{c} Var(lnRR_{+})=\frac{1}{\sum_{i=1}^{k}w_{i}} \end{array}$$

(6) $$\begin{array}{c} 95\%CI=lnRR_{+}\pm 1.96\sqrt{Var(lnRR_{+})} \end{array}$$

For ease of interpretation of the analysis, weighted effect sizes were converted to percentage changes (%) using Equation 7 based on comparisons between the experimental and control groups.

(7) $$\begin{array}{c} percentage\;change(\%)=(e^{lnRR_{+}}-1)\times 100\% \end{array}$$

### 2.3 Data-analysis

Meta-analyses, statistical analyses and plots were performed in R (version 4.4.1) software. Response ratios were calculated using a random-effects model with restricted maximum likelihood (RMEL) to calculate weighted response ratios and heterogeneity between the data pairs (I^2^) (Wang et al., 2024). We used random forest to examine the relative importance of each predictor variable to the response ratio. The potential for publication bias to influence the outcomes of meta-analyses necessitates the implementation of fail-safe analyses to identify any such bias (Du et al., 2023). The results demonstrate that rosenthal > 5N+10 (N representing the sample number) and I^2^ are greater than 95% (**Supplementary Table 1**). This indicates that this study was not affected by publication bias and that the conclusions are reliable.

## 3 Results

### 3.1 Overall effects of eCO_2_ on cucumbers

**Figure 2:**
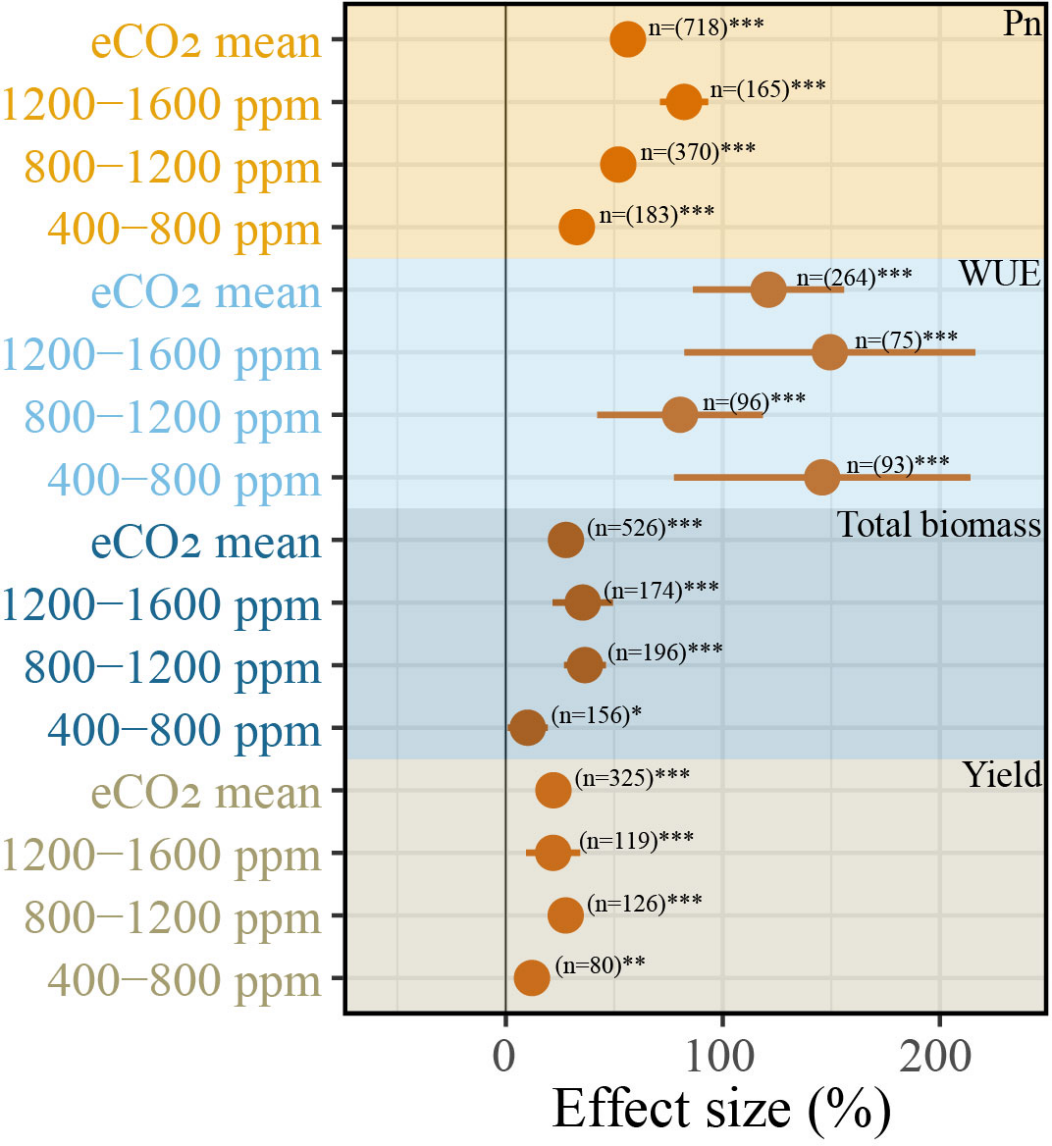
Overall response rate of cucumber photosynthesis, water use efficiency, total biomass and yield to eCO_2_. The points represent the mean values and the numbers next to them indicate the cumulative sample number of studies. Error lines represent 95% CIs. Asterisks (*) indicate significant differences (*p < 0.05, **p < 0.01, ***p < 0.001).

The analysis of the data indicates that high-concentration carbon dioxide treatment significantly enhances the net photosynthetic rate and yield of cucumbers, with respective increases of 56.31% (95% CI: 50.31% to 62.32%) and 21.98% (95% CI: 16.60% to 27.36%). Concurrently, water use efficiency and total biomass accumulation exhibited varying degrees of improvement, increasing by 121.11% (95% CI: 86.26% to 155.96%) and 27.74% (95% CI: 20.88% to 34.61%), respectively (**Figure 2**). The CO_2_ concentration range of 1200–1600 ppm was found to be more favorable for photosynthesis and water use efficiency, whereas the 800–1200 ppm range was more conducive to total biomass and yield enhancement (**Supplementary Table 2**).

### 3.2 Response of photosynthesis to environmental factors under eCO_2_

**Figure 3:**
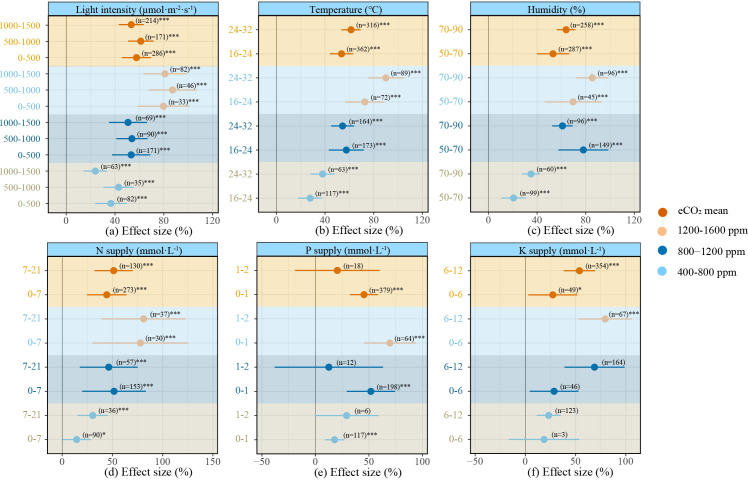
Effect of various environmental factors on cucumber pn under eCO_2_. The points represent the mean values and the numbers next to them indicate the cumulative sample number of studies. Error lines represent 95% CIs. Asterisks (*) indicate significant differences (*p < 0.05, ***p < 0.001). The same as above.

In comparison with standard carbon dioxide concentrations, the net photosynthetic rate of cucumbers demonstrated an upward trend in response to eCO_2_ (**Supplementary Figure 2a**). An analysis of environmental factors under eCO_2_ conditions indicated that enhancing light intensity, temperature, humidity, and nitrogen-potassium supply proved more effective in boosting net photosynthetic rates, particularly within the 1200–1600 ppm concentration range (**Figure 3**). A random forest analysis of eCO_2_–environmental factor interaction weights identified temperature, light intensity, and potassium supply as key regulatory factors (**Supplementary Figure 3a**). Subsequent analysis revealed that light intensity and temperature exhibited an initial increase followed by a decline, whereas increased nitrogen supply resulted in a sustained rise in net photosynthetic rate (**Supplementary Figure 4**).

### 3.3 Response of WUE to environmental factors under eCO_2_

**Figure 4:**
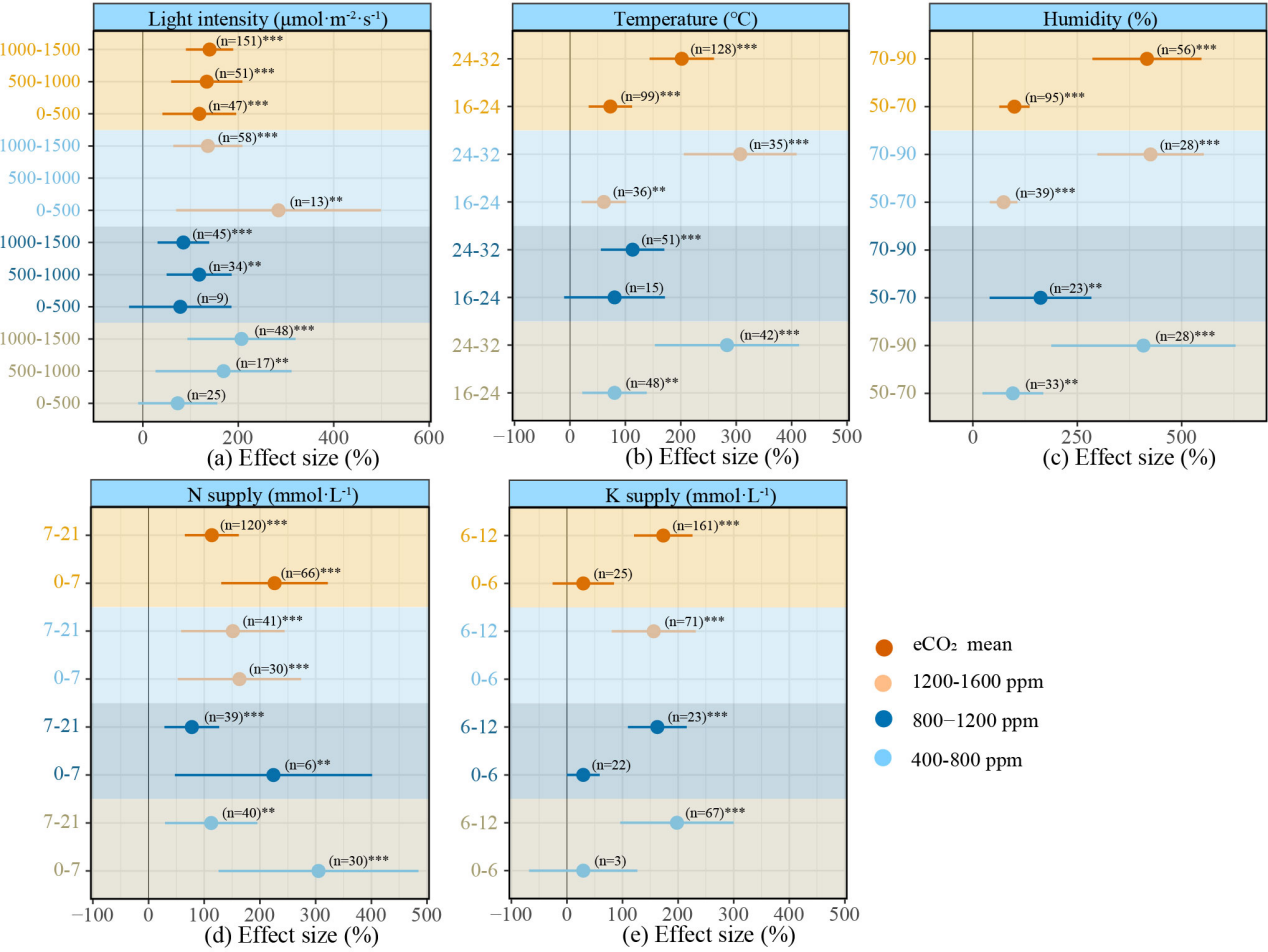
Effect of various environmental factors on cucumber WUE under eCO_2_. The points represent the mean values and the numbers next to them indicate the cumulative sample number of studies. Error lines represent 95% CIs. Asterisks (*) indicate significant differences (**p < 0.01, ***p < 0.001). The same as above.

The regulation of cucumber water use efficiency by eCO_2_ exhibited a pattern of initial decline followed by increase, indicating heightened uncertainty in WUE responses under excessively elevated CO_2_ conditions (**Supplementary Figure. 2b**). The analysis of CO_2_ gradient stratification demonstrated that eCO_2_ enrichment significantly amplified the regulatory effects of key factors. Under eCO_2_ conditions, the positive effects of high light intensity, moderate temperature, high humidity, and high potassium supply on WUE were markedly enhanced (**Figure 4**). Among the key regulatory factors, temperature, nitrogen supply, and light intensity exerted the strongest influence, emerging as the core drivers of WUE variation under eCO_2_. (**Supplementary Figure. 3b**) An enhancement in light intensity and temperature exerted a positive regulatory effect on water use efficiency under eCO_2_, though this effect diminished at excessively high levels. Furthermore, the application of nitrogen fertilizer appeared to exert a persistent negative regulatory influence (**Supplementary Figure 5**).

### 3.4 Response of total biomass to environmental factors under eCO_2_

**Figure 5:**
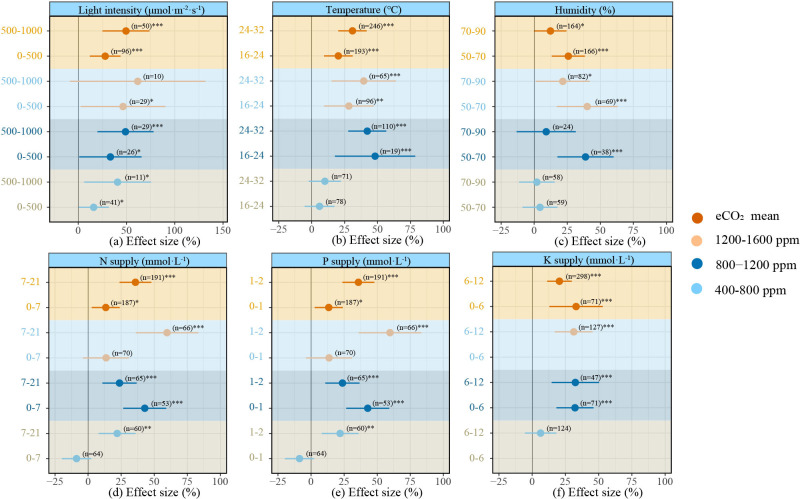
Effect of various environmental factors on cucumber biomass accumulation under eCO_2_. The points represent the mean values and the numbers next to them indicate the cumulative sample number of studies. Error lines represent 95% CIs. Asterisks (*) indicate significant differences (*p < 0.05, **p < 0.01, ***p < 0.001). The same as above.

As the levels of carbon dioxide increased, the cucumber biomass accumulation exhibited a synchronous growth trend (**Supplementary Figure 2c**). A detailed analysis of the interactions between eCO_2_ and environmental variables was conducted, revealing that higher light intensity and temperature interacted with varying eCO_2_ levels to exert a more pronounced effect on biomass growth. Conversely, humidity exhibited an opposite effect (**Figure 5**). In addition to potassium fertilization, increased nitrogen and phosphorus inputs also promoted biomass accumulation, particularly at 1200–1600 ppm concentrations. Weighted analyses revealed temperature, nitrogen supply, and humidity as key regulatory factors influencing biomass accumulation (**Supplementary Figure 3c**). In the presence of elevated levels of carbon dioxide (eCO_2_), there was a demonstrable increase in the biomass, which was observed to rise in proportion to rising light intensity, temperature, and nitrogen supply. Concurrently, there was a decline in humidity, phosphorus, and potassium supply (**Supplementary Figure 6**).

### 3.5 Response of yield to environmental factors under eCO_2_

**Figure 6:**
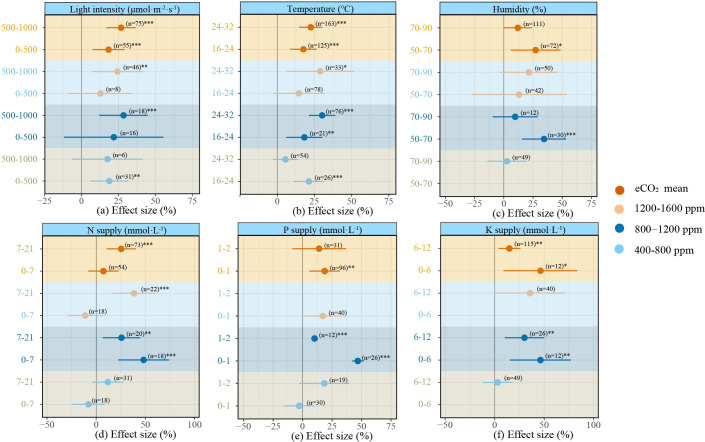
Effect of various environmental factors on cucumber yield under eCO_2_. The points represent the mean values and the numbers next to them indicate the cumulative sample number of studies. Error lines represent 95% CIs. Asterisks (*) indicate significant differences (*p < 0.05, **p < 0.01, ***p < 0.001). The same as above.

It is evident that the range of 800–1200 ppm CO_2_ concentration is most conducive to enhancing cucumber yield (**Supplementary Figure 2d**). In the context of vegetable cultivation under eCO_2_ conditions, it has been observed that other environmental factors also exert varying degrees of influence on vegetable production (Dong et al., 2020). This meta-analysis confirms that the impact of eCO_2_ on cucumber yield varies significantly with changes in light intensity, temperature, humidity, and nitrogen-phosphorus-potassium supply levels (**Figure 6**). The interaction between eCO_2_ and environmental factors, including temperature, humidity, and light intensity, was found to be crucial in regulating cucumber yield (**Supplementary Figure 3d**). It is observed that an increase in light intensity, temperature, and nitrogen supply results in a more pronounced increase in cucumber yield. In contrast, an excess of humidity and phosphorus-potassium supply has been demonstrated not to yield this effect (**Supplementary Figure 7**). In addition, although no significant differences were observed, cucumber yield exhibited a negative response to lower nitrogen and phosphorus supply at carbon dioxide concentrations between 400–800 ppm.

## 4 Discussion

### 4.1 The effect of eCO_2_ on cucumbers

**Figure 7:**
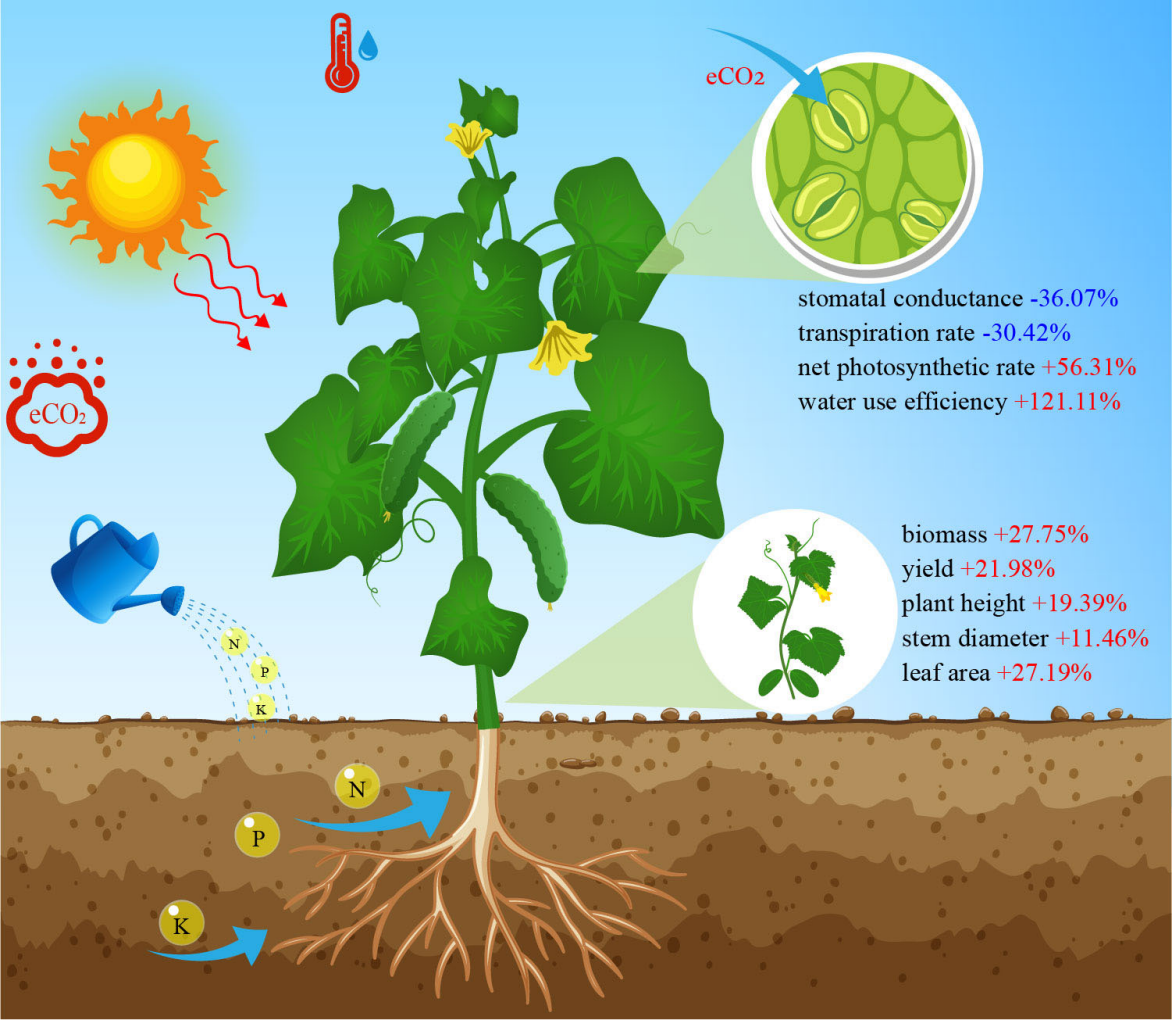
Schematic representation outlining changes in cucumber growth, photosynthesis and yield under eCO_2_.

Increases in atmospheric carbon dioxide concentrations have the potential to positively impact agricultural production, particularly in the future. A comprehensive understanding of the growth response of cucumbers to eCO_2_ is imperative for sustainable development. This meta-analysis demonstrates that elevated carbon dioxide concentrations significantly increase cucumber plant height, stem diameter and leaf area (**Supplementary Figure 8**), exhibiting a strong positive correlation (**Supplementary Figure 9**). It is evident that eCO_2_ exerts a favorable influence on the growth and yield of cucumbers. It has been demonstrated that an increase in carbon dioxide concentrations results in elevated levels of carbon dioxide in plant leaves, thus enhancing photosynthetic efficiency. Plants are able to convert carbon dioxide into organic compounds such as sugars more quickly, providing more energy and nutrients for plant growth (Sugiura et al., 2024). At the same time, increased carbon dioxide concentrations lead to a decrease in stomatal conductance, reducing transpiration and water loss, and thereby improving water use efficiency (Robredo et al., 2007; Osman et al., 2024). The enhancement in photosynthesis and water use efficiency observed in cucumbers cultivated under elevated CO_2_ conditions is the primary factor contributing to the substantial increases in plant height, stem diameter, leaf area, and yield (**Figure 7**).

Research indicates that eCO_2_ can enhance photosynthetic efficiency, enabling plants to fix more carbon and thereby increase biomass (Qiang et al., 2020; Hou et al., 2021). As the levels of carbon dioxide in the atmosphere increase, there is a concomitant and significant increase in the overall biomass accumulation of cucumbers. However, at carbon dioxide concentrations of 400–800 ppm, this effect appears to be less favorable for stem biomass accumulation (**Supplementary Figure 8**). This finding suggests that the promotional effect may be subject to variation and may be influenced by various factors, including plant species, growth conditions, and the specific experimental design (Maschler et al., 2022). Whilst acknowledging the constant interplay between eCO_2_ and other environmental factors, it is vital to recognize that these interactions do not inherently negate the overall promotional effect of eCO_2_ on cucumbers. It is important to note that slight variations in the effects observed under different environmental conditions may be attributable to these interacting factors.

### 4.2 The effects of light intensity with eCO_2_ on cucumbers

The effects of CO_2_ enrichment on plants are not independent of other environmental factors, but rather interact with them. In the context of CO_2_ enrichment, it has been demonstrated that the net photosynthetic rate of cucumbers can be enhanced by a variety of light intensities. The net photosynthetic rate of cucumbers attains its zenith under conditions of CO_2_ concentrations ranging from 1200–1600 ppm, in conjunction with light intensities spanning from 500-1000 μmol·m^-2^·s^-2^. This phenomenon can be attributed to the capacity of elevated CO_2_ levels to elevate the light saturation point of plants. In the presence of optimal light intensity, higher levels of light energy conversion efficiency are exhibited by plants. Nonetheless, in conditions of low light intensity or intense light conditions, insufficient supplies of ATP and NADPH act as a limiting factor in the enhancement of net assimilation efficiency (Xin et al., 2019). An increase in light intensity and carbon dioxide concentration has been shown to progressively enhance growth parameters such as fresh weight, dry weight and leaf area. The interaction between light intensity and carbon dioxide concentration can modify biomass distribution during growth (Hosseinzadeh et al., 2021). The findings of the present study demonstrate that eCO_2_ and high light intensity significantly increased the total biomass and yield of cucumbers. Furthermore, it was observed that eCO_2_ had a more pronounced effect on root development (**Supplementary Figure 8**).

In conditions that are conducive to photosynthesis, an increase in the concentration of carbon dioxide, in conjunction with an escalation in light intensity, has been observed to exert a substantial influence on the growth parameters of plants, including plant height, leaf area, and biomass (Pan et al., 2020). The present study demonstrates that the yield of cucumber is significantly increased under eCO_2_ conditions, especially at higher light intensities. This phenomenon is attributed to the efficient flow of carbon between source and sink compartments, facilitated by the addition of carbon dioxide and increased light intensity. The effective photosynthetic radiation in greenhouses is significantly lower than in the external environment. Consequently, the integration of supplemental carbon dioxide with augmented light intensity is poised to emerge as a pivotal strategy for augmenting crop yield and quality (Prakash et al., 2023; Kim et al., 2025).

### 4.3 The effect of temperature with eCO_2_ on cucumbers

Temperature exerts a regulatory influence on the growth rate and morphogenesis of plants, with this influence being exerted through the medium of the activity of enzymes related to photosynthesis, the photorespiration rate, and the efficiency of carbon allocation (Rachmilevitch et al., 2006; Amaral et al., 2024). Within the appropriate temperature range, increased carbon dioxide concentrations typically enhance photosynthetic rates and promote biomass accumulation (Sharma et al., 2020). However, at excessively high temperatures, photosynthesis may be inhibited, altering the composition of secondary metabolites, which may be difficult to restore even under high carbon dioxide concentrations (Roy et al., 2024). Conversely, low temperatures may lead to cellular damage, metabolic slowdown, and growth stagnation (Cao et al., 2024). A meta-analysis of cucumbers cultivated under conditions of elevated carbon dioxide concentrations has demonstrated that net photosynthetic rates exhibit a significant increase in response to rising temperatures, thereby promoting biomass and yield accumulation. As a thermophilic plant, cucumbers exhibit increased photosynthetic rates with rising temperatures within a certain temperature range. Additionally, as carbon dioxide concentrations rise, the optimal photosynthetic temperature for crops also increases (Nilsen et al., 1983). Overall, both eCO_2_ and elevated temperatures have positive effects on cucumber biomass accumulation and yield. Surprisingly, we found that at carbon dioxide concentrations of 400–800 ppm, higher temperatures (24–32 °C) had a less pronounced effect on yield enhancement compared to lower temperatures (**Figure 6**). A comparison of the available data sets revealed a positive correlation between yield and both warming and eCO_2_ for the majority of crops, whilst the yield of corn was found to be negatively impacted (Shin et al., 2022; Zhu et al., 2023). Contrary to expectations, the findings revealed that at carbon dioxide concentrations ranging from 400–800 ppm, elevated temperatures (24–32 C) exerted a comparatively diminished influence on the enhancement of cucumber yield in comparison to reduced temperatures. This may be because the eCO_2_ level is not high enough, so even though photosynthetic rates and total biomass accumulation increase at higher temperatures, accelerated phenological shifts and source-sink imbalances prevent photosynthetic products from being effectively transported to economically productive parts of the plant. However, the overall impact of eCO_2_ and elevated temperature levels on cucumber biomass accumulation and yield is positive, with these factors playing a significant role in the regulation of photosynthesis, growth, and yield. This indicates that they remain a highly reliable regulatory strategy in production.

### 4.4 The effect of humidity with eCO_2_ on cucumbers

The primary function of humidity is to regulate transpiration rates and the rate at which CO_2_ enters leaves by affecting stomatal conductance. Moderate increases in humidity have been demonstrated to promote stomatal opening and increase carbon dioxide absorption, thereby improving photosynthetic efficiency (Urban et al., 2017). Higher humidity at eCO_2_ increased the net photosynthetic rate and WUE of cucumbers, but we found that this was not the most favorable combination for biomass accumulation and yield improvement. eCO_2_ and higher humidity increased the net photosynthetic rate of cucumbers, but we found that this was not the most favorable combination for biomass accumulation and yield improvement. While higher humidity levels have been demonstrated to enhance water status and augment the net photosynthetic rate, they have also been observed to exert deleterious effects. On the one hand, excessive humidity reduced plant transpiration rate, affecting nutrient absorption and transport (McDonald et al., 2002), and even causing stress to plants when combined with high temperatures (Cornish et al., 2025). the other hand, high humidity may also increase the risk of plant diseases, further affecting plant health and yield (Li et al., 2022). Consequently, the process of enriching carbon dioxide with the objective of enhancing productivity ought to be executed with meticulous humidity regulation.

### 4.5 The effect of fertilization with eCO_2_ on cucumbers

Fertilizers play a pivotal role in facilitating the optimal growth and development of vegetables, with particular emphasis on essential nutrients such as nitrogen, phosphorus, and potassium. In high-concentration CO_2_ environments, the nutrient use efficiency of plants undergoes changes (Cui et al., 2023). Nitrogen is a pivotal component of chlorophyll and photosynthetic enzymes. An adequate N supply is imperative for sustaining photosynthetic efficiency under conditions of CO_2_ enrichment, and can substantially augment photosynthetic rates and biomass under such conditions (Hazra et al., 2019). It is evident that an increase in carbon dioxide concentrations exerts a substantial influence on the photosynthetic rates, biomass accumulation, and yield of cucumbers, when different nitrogen fertilizer supplies are employed. A substantial increase in the photosynthetic rate, biomass accumulation, and yield of cucumbers was observed in experiments conducted with higher nitrogen supply levels. However, the supply of low nitrogen had a minimal promotional effect on cucumbers under eCO_2_ conditions, and even inhibited biomass accumulation at carbon dioxide concentrations of 400–800 ppm. Furthermore, at concentrations ranging from 400–800 ppm and from 1200–1600 ppm, an inhibitory effect on yield was observed. This is because although eCO_2_ usually stimulates carbon assimilation, it can also lead to a decrease in nutrient concentrations in plant tissues, especially nitrogen (Zhao et al., 2021). In instances where the supply of nitrogen fertilizer is inadequate, the promoting effect of elevated carbon dioxide concentrations on plant growth is counteracted (Reich et al., 2014).

Phosphorus is imperative for energy transfer, root development, and flower and fruit formation, while potassium is involved in regulating stomatal opening and closing, enzyme activation, and water relations (Gu et al., 2024; Jan et al., 2024). Increased carbon dioxide concentrations can reduce the accumulation of phosphorus, an essential element for plants (Bouain et al., 2022). In circumstances where phosphorus supply is inadequate, elevated carbon dioxide concentrations have been demonstrated to exert a deleterious effect on photosynthesis, potentially weakening or even preventing it from occurring (Pandey et al., 2015). Increased carbon dioxide concentrations can reduce the accumulation of phosphorus, an essential element for plants. When phosphorus fertilizer supply is insufficient, the promotional effect of elevated carbon dioxide concentrations on plant growth may weaken or even inhibit photosynthesis. In summary, under eCO_2_ conditions, the application of an appropriate phosphorus fertilizer concentration in the nutrient solution has been demonstrated to promote cucumber photosynthesis and yield, with higher phosphorus concentrations having a significant effect on biomass accumulation. It is noteworthy that within the range of 800–1200 ppm of carbon dioxide and a phosphorus content in the nutrient solution that corresponds to the standard value (1 mmol·L^-1^), there is a promotion of approximately 50% in the photosynthesis, biomass, and yield of cucumbers. Potassium plays a pivotal role in plants by regulating osmosis, activating enzymes, and controlling stomatal movement. By influencing photosynthesis, it ensures the normal process of carbon assimilation, thereby regulating plant growth and yield (Ho et al., 2020). Although higher potassium fertilizer concentrations can significantly increase cucumber photosynthetic rates, their promotional effects on biomass and yield are less pronounced than those of conventional nutrient solution concentrations. This may be because high-potassium nutrient solutions interfere with plants’ absorption and utilization of other essential elements, leading to nutrient imbalance and consequently reducing plant biomass and yield (Ho et al., 2020).

### 4.6 Limitations of the study—future research

The present meta-analysis examined the response of cucumber photosynthesis, growth and yield under eCO_2_. However, the availability of eCO_2_ data was limited, and no detailed gradient interaction analysis was conducted on the effects of eCO_2_ and other environmental factors (light intensity, temperature, etc.) on cucumbers. Furthermore, the paucity of data precluded analysis of the effects of eCO_2_ on cucumber quality, a subject that demands further research in the future. Consequently, further data is required to facilitate precise predictions regarding the impact of eCO_2_ on cucumber growth and yield in the context of global climate change and greenhouse conditions characterized by microclimates.

## 5 Conclusion

The findings of this study suggest that the promotion of photosynthesis, growth, and yield in cucumbers under eCO_2_ conditions is of considerable significance. The findings of this study demonstrate that eCO_2_ have a positive impact on net photosynthesis, biomass, and yield, with an observed increase of 56.31%, 27.75%, and 21.98%, respectively. The optimal effect was observed within a concentration range of 800–1200 ppm. Furthermore, for eCO_2_, it has been demonstrated that elevated light intensity, temperature, and adequate humidity and fertilizer supply interact synergistically to create optimal conditions for cucumber production. The present study analyzed the growth response of cucumbers under eCO_2_, which will provide a more informed theoretical framework for environmental regulation in cucumber cultivation and contribute to green, sustainable development.

## Data Availability

The original contributions presented in the study are included in the article/**Supplementary Material**. Further inquiries can be directed to the corresponding author.
