# Supplementary material for: Optimizing carbon dioxide enrichment with environmental management to improve photosynthesis, water use efficiency and yield in cucumbers: a meta-analysis study
Source: Front Plant Sci. 2026 May 28;17:1826536. doi: 10.3389/fpls.2026.1826536 (PMC13254091; doi:10.3389/fpls.2026.1826536)
Supplement: Supplementary file 1 [file Table1.docx]

Supplementary Material

Supplementary Table 1. Total heterogeneity (Qtotal), probability (p) value, I^2^, and publication bias of each variable.

| **Variables** | **Q_total_** | **p-values** | **I^2^** | **Fail-safe numbers** | **Publication bias** |
| --- | --- | --- | --- | --- | --- |
| Yield | 2185.47 | ＜0.0001*** | 98.59% | 81878 | NO |
| Plant height | 2670.30 | ＜0.0001*** | 99.63% | 19932 | NO |
| Stem diameter | 539.08 | ＜0.0001*** | 97.04% | 7149 | NO |
| Leaf area | 2668.06 | ＜0.0001*** | 99.27% | 38872 | NO |
| Biomass (Total) | 6274.65 | ＜0.0001*** | 99.11% | 240150 | NO |
| Biomass  (root) | 2866.67 | ＜0.0001*** | 98.99% | 44815 | NO |
| Biomass (stem) | 1205.45 | ＜0.0001*** | 99.35% | 3760 | NO |
| Biomass  (leaf) | 1419.76 | ＜0.0001*** | 99.19% | 8022 | NO |
| Pn | 20819.72 | ＜0.0001*** | 99.56% | 2850252 | NO |
| Gs | 10123.53 | ＜0.0001*** | 99.87% | 63035 | NO |
| Tr | 9483.55 | ＜0.0001*** | 99.68% | 28584 | NO |
| WUE | 81116.65 | ＜0.0001*** | 99.94% | 2312929 | NO |

Supplementary Table 2. The results of the ANOVA showed the effect of eCO_2_ of 400-800, 800-1200 and 1200-1600 ppm on the photosynthesis, growth and yield response of cucumber.

| **Variables** | **Df** | **Sum Sq** | **Mean Sq** | **F** | ***p*-values** | **eCO_2_** | **Mean, [95% Confidence Interval] (%)** |
| --- | --- | --- | --- | --- | --- | --- | --- |
| Yield | 2 | 0.1980 | 0.9899 | 2.642 | 0.0439* | 1200-1600 ppm | 21.84 [9.40 to 34.28] b |
|  |  |  |  |  |  | 800-1200 ppm | 27.63 [20.91 to 34.36] a |
|  |  |  |  |  |  | 400-800 ppm | 12.09 [3.94 to 20.24] c |
| Plant height | 2 | 0.3366 | 0.1683 | 5.023 | 0.0147* | 1200-1600 ppm | 56.67 [13.44 to 99.91] a |
|  |  |  |  |  |  | 800-1200 ppm | 15.10 [7.58 to 22.61] b |
|  |  |  |  |  |  | 400-800 ppm | 10.38 [-3.36 to 24.13] b |
| Stem diameter | 2 | 0.0314 | 0.0157 | 2.325 | 0.0124* | 1200-1600 ppm | 18.90 [2.46 to 35.34] a |
|  |  |  |  |  |  | 800-1200 ppm | 10.58 [6.97 to 14.19] b |
|  |  |  |  |  |  | 400-800 ppm | 4.08 [2.38 to 5.78] c |
| Leaf area | 2 | 0.2100 | 0.1050 | 2.902 | 0.0272* | 1200-1600 ppm | 56.74 [17.03 to 96.44] a |
|  |  |  |  |  |  | 800-1200 ppm | 24.25 [5.55 to 42.95] ab |
|  |  |  |  |  |  | 400-800 ppm | 23.38 [15.56 to 31.20] b |
| Biomass (total) | 2 | 0.908 | 0.4538 | 6.736 | 0.0019** | 1200-1600 ppm | 35.49 [21.56 to 49.42] a |
|  |  |  |  |  |  | 800-1200 ppm | 36.49 [26.85 to 46.14] a |
|  |  |  |  |  |  | 400-800 ppm | 10.12 [0.94 to 19.30] b |
| Biomass (root) | 2 | 0.991 | 0.4953 | 6.245 | 0.0038** | 1200-1600 ppm | 41.91 [23.85 to 59.96] a |
|  |  |  |  |  |  | 800-1200 ppm | 30.93 [16.06 to 45.81] a |
|  |  |  |  |  |  | 400-800 ppm | 4.64 [-5.68 to 14.97] b |
| Biomass (stem) | 2 | 0.58 | 0.2899 | 2.817 | 0.0348* | 1200-1600 ppm | 20.24 [4.34 to 36.13] ab |
|  |  |  |  |  |  | 800-1200 ppm | 39.88 [3.86 to 75.89] a |
|  |  |  |  |  |  | 400-800 ppm | -1.14 [-10.52 to 8.24] b |
| Biomass (leaf) | 2 | 0.099 | 0.0450 | 0.444 | 0.3833 | 1200-1600 ppm | 27.24 [4.55 to 49.94] a |
|  |  |  |  |  |  | 800-1200 ppm | 28.96 [4.94 to 52.97] a |
|  |  |  |  |  |  | 400-800 ppm | 12.63 [-0.26 to 25.51] a |
| Pn | 2 | 1.833 | 0.9167 | 22.72 | ＜0.0001*** | 1200-1600 ppm | 82.19 [71.14 to 93.25] a |
|  |  |  |  |  |  | 800-1200 ppm | 51.88 [44.01 to 59.75] b |
|  |  |  |  |  |  | 400-800 ppm | 32.78 [25.83 to 39.72] c |
| Gs | 2 | 0.684 | 0.3421 | 2.79 | 0.0025* | 1200-1600 ppm | -44.47 [-63.60 to -25.35] a |
|  |  |  |  |  |  | 800-1200 ppm | -25.15 [-35.14 to -15.16] b |
|  |  |  |  |  |  | 400-800 ppm | -39.68 [-61.33 to -18.03] ab |
| Tr | 2 | 0.509 | 0.2544 | 1.093 | 0.2461 | 1200-1600 ppm | -32.93 [-47.03 to -18.84] a |
|  |  |  |  |  |  | 800-1200 ppm | -20.40 [-33.46 to -7.34] a |
|  |  |  |  |  |  | 400-800 ppm | -37.77 [-53.21 to -22.33] a |
| WUE | 2 | 1.031 | 0.5156 | 1.533 | 0.0228* | 1200-1600 ppm | 149.38 [82.32 to 216.43] a |
|  |  |  |  |  |  | 800-1200 ppm | 80.33 [42.16 to 118.49] ab |
|  |  |  |  |  |  | 400-800 ppm | 145.84 [77.54 to 214.14] a |


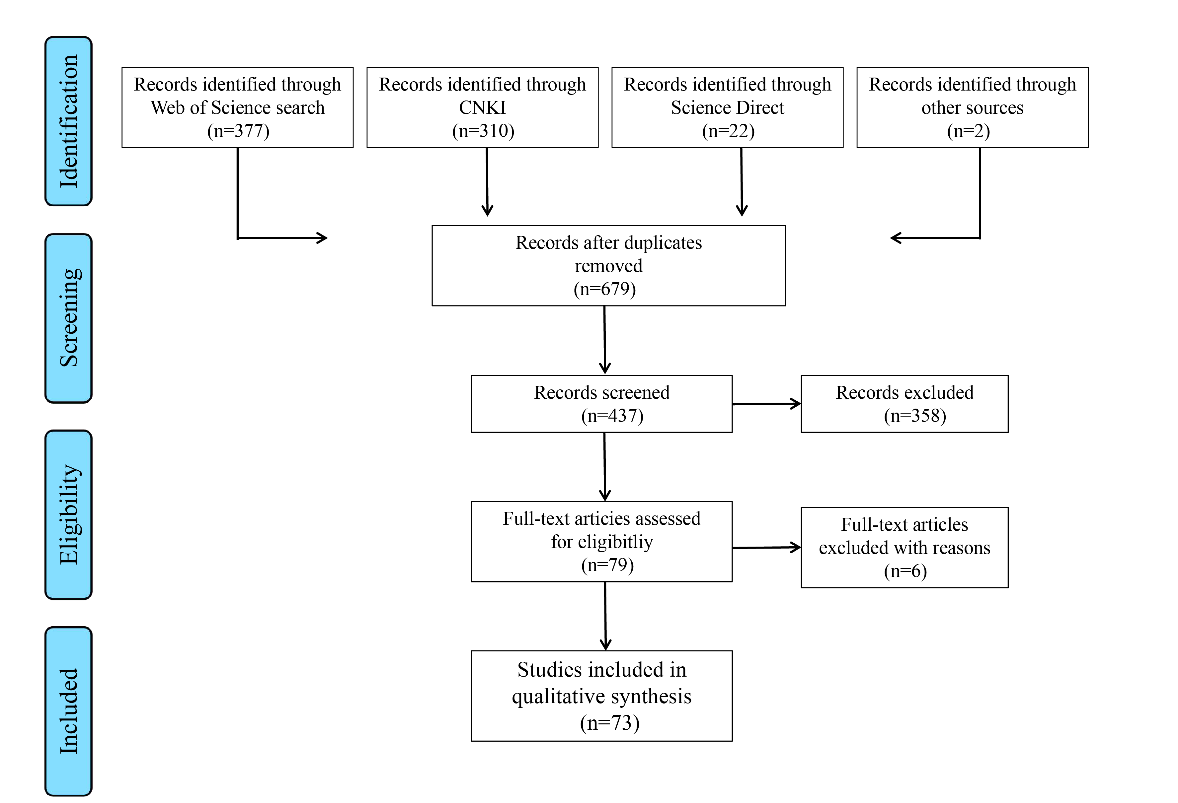


**Supplementary Figure 1.** Analytical flowchart of inclusion and exclusion criteria.


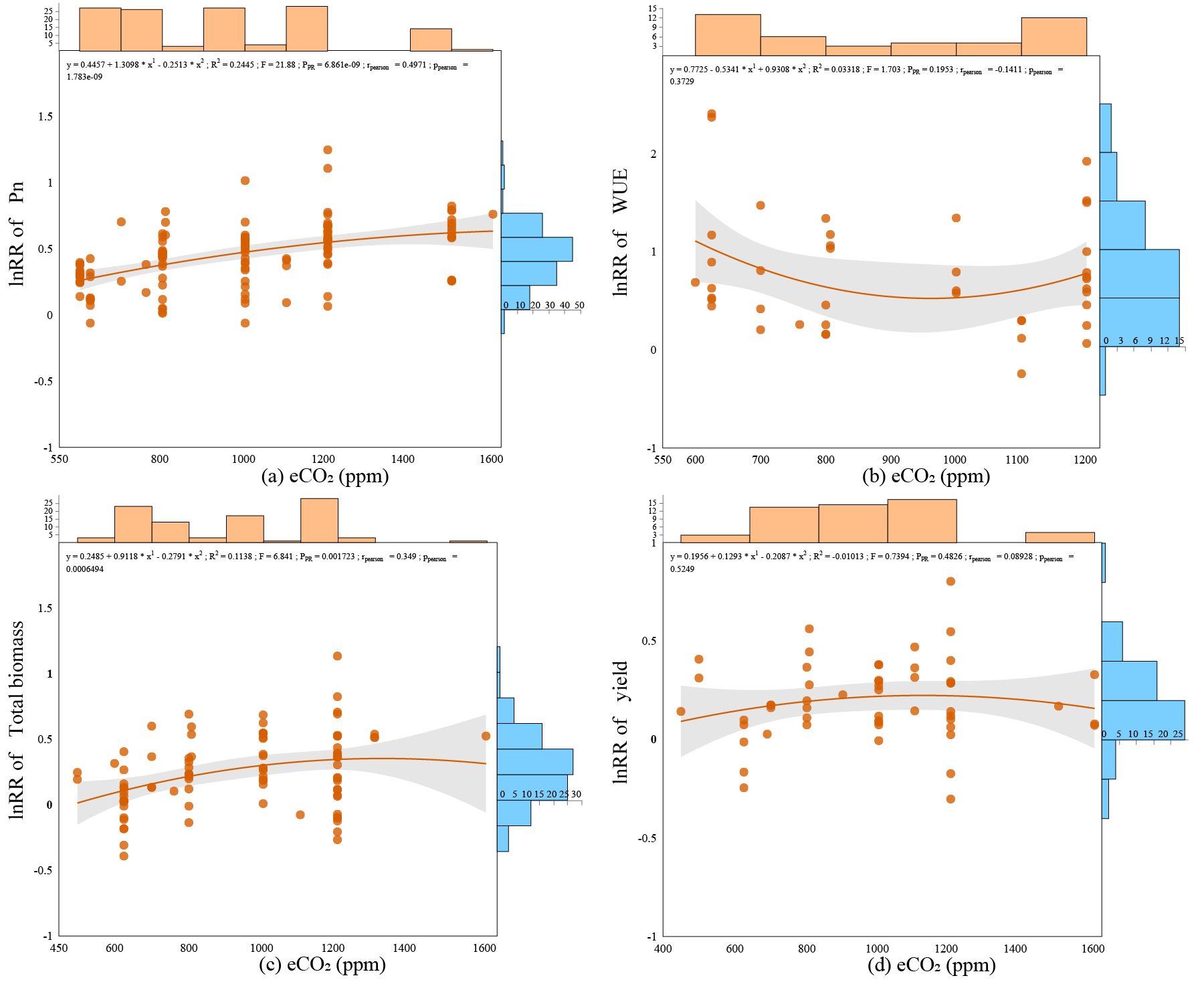


**Supplementary Figure 2.** Correlation of CO_2_ concentration with cucumber Pn (a), WUE (b), total biomass (c) and yield (d) in response to eCO_2_.

**
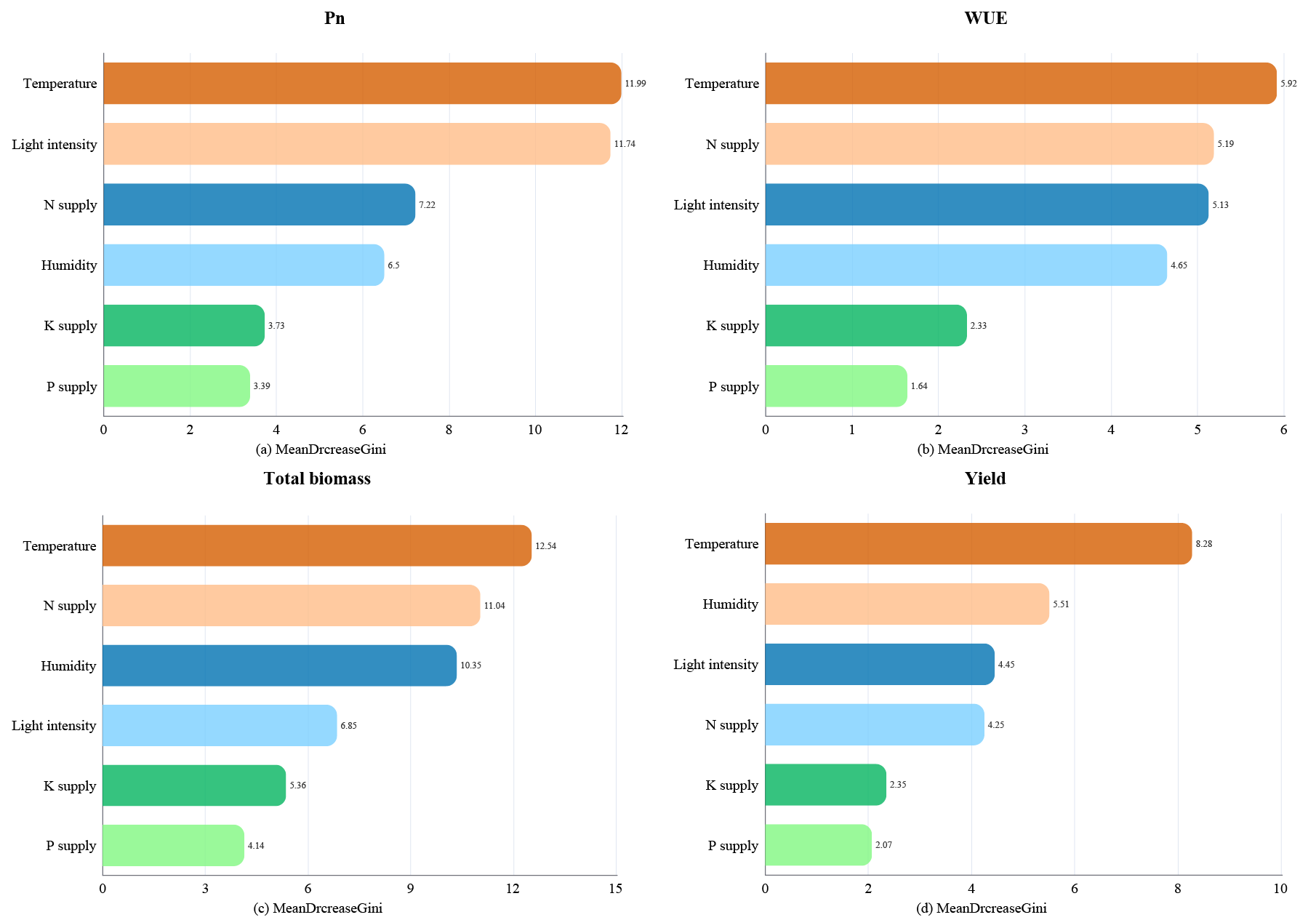
**

**Supplementary Figure 3.** Importance of various environmental predictor variables for cucumber Pn (a), WUE (b), total biomass (c) and yield (d).


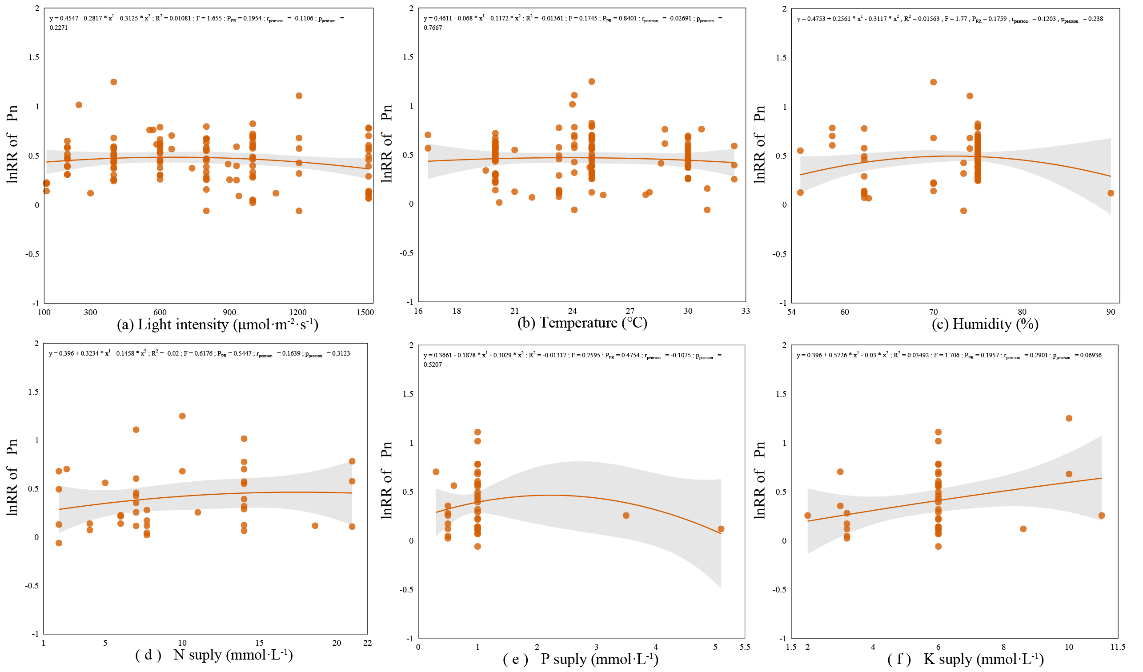


**Supplementary Figure 4.** Correlation of cucumber Pn with predicted environmental variables light intensity (a), temperature (b), humidity (c), N supply (d), P supply (e) and K supply (f) in response to eCO_2_.


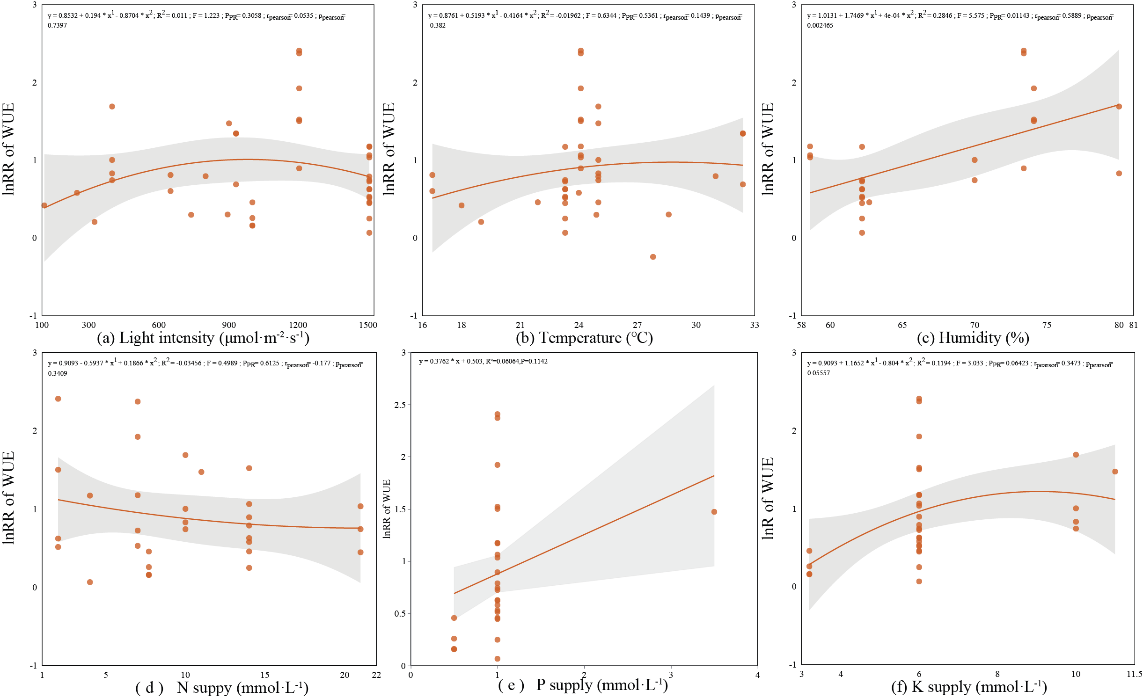


**Supplementary Figure 5.** Correlation of cucumber WUE with predicted environmental variables light intensity (a), temperature (b), humidity (c), N supply (d), P supply (e) and K supply (f) in response to eCO_2_. The same as above.


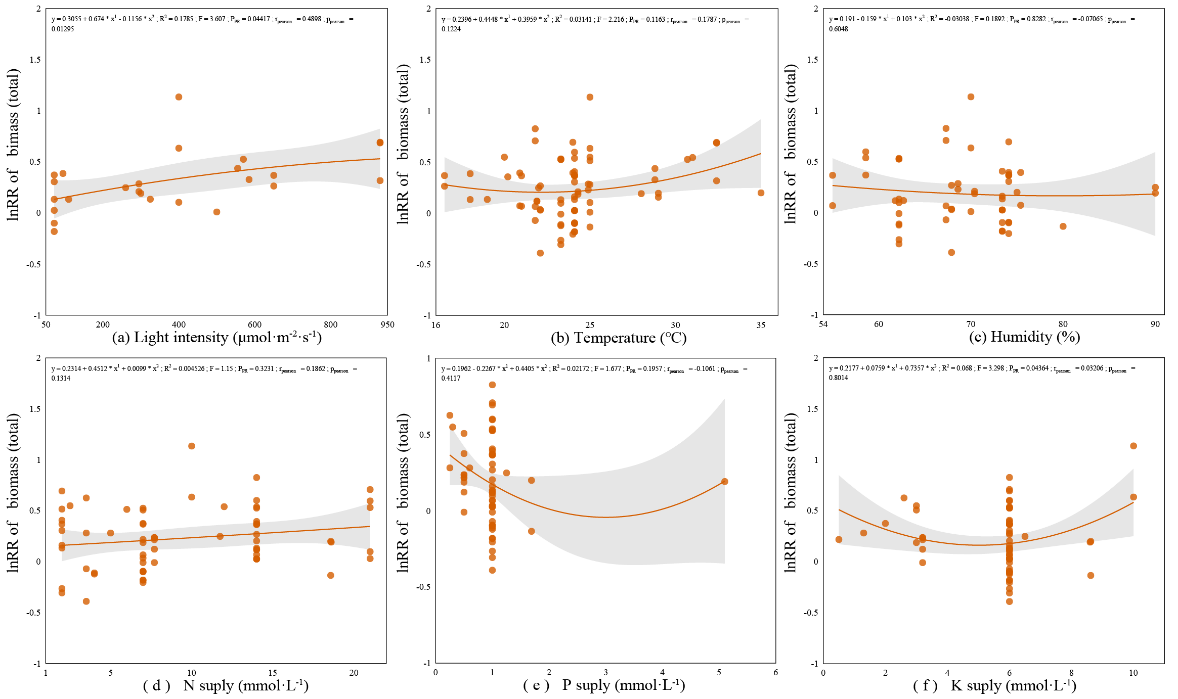


**Supplementary Figure 6.** Correlation of cucumber total biomass with predicted environmental variables light intensity (a), temperature (b), humidity (c), N supply (d), P supply (e) and K supply (f) in response to eCO_2_. The same as above.


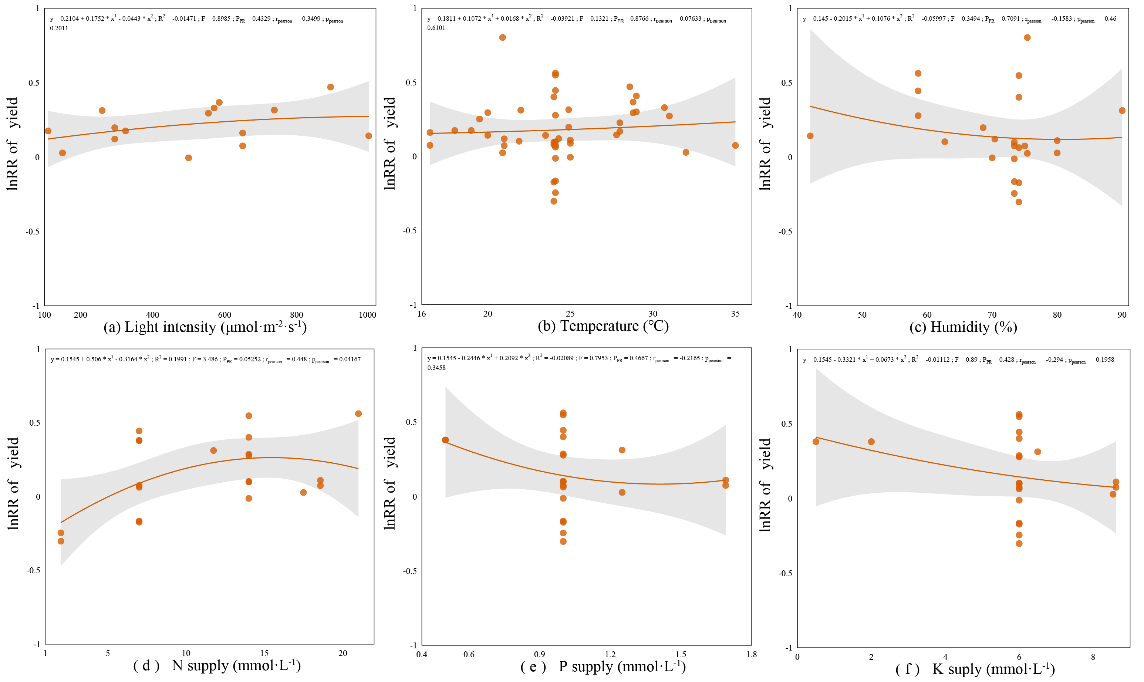


**Supplementary Figure 7.** Correlation of cucumber yield with predicted environmental variables light intensity (a), temperature (b), humidity (c), N supply (d), P supply (e) and K supply (f) in response to eCO_2_. The same as above.


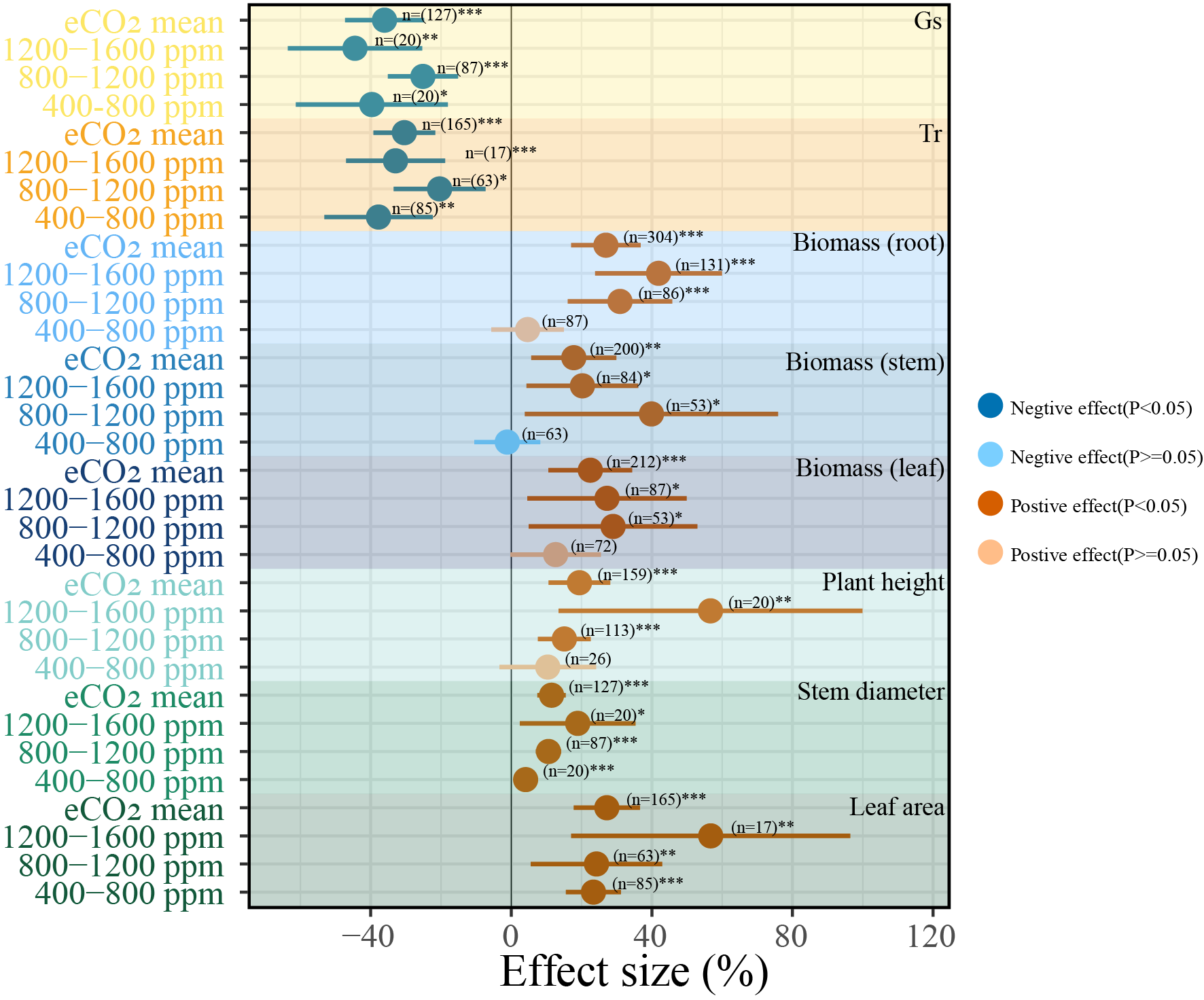


**Supplementary Figure 8.** Overall response rate of cucumber Gs, Tr, biomass (root), biomass (stem), biomass (leaf), plant height, stem diameter and leaf area to eCO_2_. The points represent the mean values and the numbers next to them indicate the cumulative sample number of studies. Error lines represent 95% CIs. Asterisks (*) indicate significant differences (*: p < 0.05, **: p < 0.01, ***: p < 0.001).


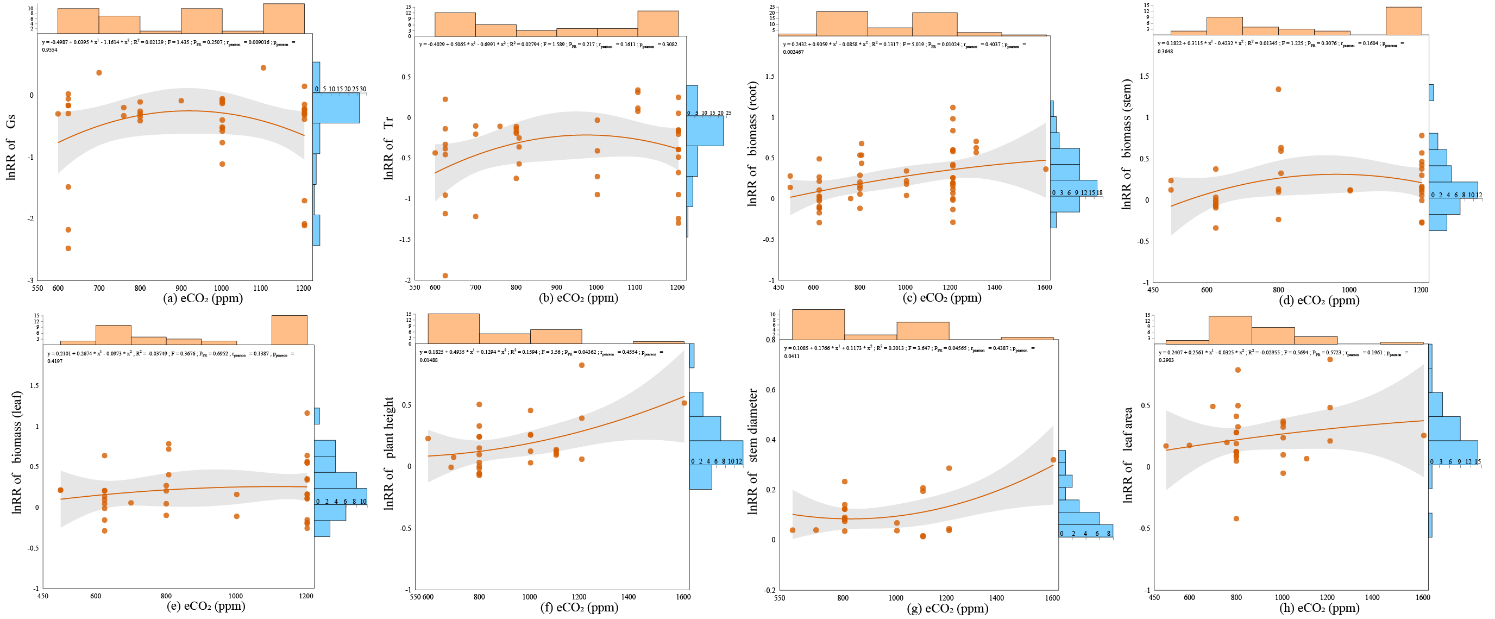


**Supplementary Figure 9.** Correlation of CO_2_ concentration with cucumber Gs (a), Tr (b), biomass (root) (c), biomass (stem) (d), biomass (leaf) (e), plant height (f), stem diameter (g) and leaf area (h) in response to eCO_2_.

**References in the database for current meta-analyses**

Abdeldaym, E. A., Hassan, H. A., El-Mogy, M. M., Mohamed, M. S., Abuarab, M. E., and Omar, H. S. (2024). Elevated concentrations of soil carbon dioxide with partial root-zone drying enhance drought tolerance and agro-physiological characteristics by regulating the expression of genes related to aquaporin and stress response in cucumber plants. *BMC Plant Biology* 24, 917. doi: 10.1186/s12870-024-05310-2

Agüera, E., Ruano, D., Cabello, P., and De La Haba, P. (2006). Impact of atmospheric CO_2_ on growth, photosynthesis and nitrogen metabolism in cucumber (Cucumis sativus L.) plants. *Journal of Plant Physiology* 163, 809–817. doi: 10.1016/j.jplph.2005.08.010

Ai, M., Liu, Z., Yang, Y., and He, L. (2005). Effects of Temperature, Light Intensity and CO_2_ Concentration on Cucumber Net Photosynthesis. *Journal of Shenyang Agricultural University* 04, 414–418.

Bao, L., Dong, J., Li, X., and Duan, Z. (2016). Effects of Elevated CO_2_, N Concentration and N Forms on Photosynthetic Pigments Concentration and Composition. *Soils* 48, 653–660.doi: 10.13758/j.cnki.tr.2016.04.005

Boese, S. R., Wolfe, D. W., and Melkonian, J. J. (1997). Elevated CO_2_ mitigates chilling‐induced water stress and photosynthetic reduction during chilling. *Plant Cell & Environment* 20, 625–632. doi: 10.1111/j.1365-3040.1997.00082x

Chen J., Li X., Wu Y., and Duan Z. (2020). Effects of selenium application on yield, selenium uptake and accumulation of cucumbers in greenhouse under elevated CO_2_ condition. *Jiangsu Journal of Agricultural Sciences* 36, 1503–1511. doi: 10.3969/j.issn.1000-4440.2020.06.021

Cui, Q., Dong, Y., Li, M., Zhang, W., Liu, B., Ai, X., et al. (2017). Effect of water-nitrogen coupling on photosynthesis and ultrastructure of cucumber leaves under CO_2_ enrichment. *Chinese Journal of Applied Ecology* 28, 1237–1245. doi: 10.1016/j.plaphy.2019.08.025

Cui, Q., Li, Y., He, X., Li, S., Zhong, X., Liu, B., et al. (2019). Physiological and iTRAQ based proteomics analyses reveal the mechanism of elevated CO_2_ concentration alleviating drought stress in cucumber (Cucumis sativus L.) seedlings. *Plant Physiology and Biochemistry* 143, 142–153. doi: 10.1016/j.plaphy.2019.08.025

Cui, Q., and Wang, J. (2004). Effects of prolonging ventilation time and enriching CO_2_ in greenhouse on the ecologic factors and photosynthetic rate of cucumber. *Chinese Journal of Eco-Agriculture* 02, 89–90. doi: 10.3724/SP.J.1011.year_id].084

Dabu, X., Li, S., Cai, Z., Ge, T., and Hai, M. (2019). The effect of potassium on photosynthetic acclimation in cucumber during CO_2_ enrichment. *Photosynthetica* 57, 640–645. doi: 10.32615/ps.2019.073

Dabu, X., Li, S., He, L., and Hai, M. (2018). Effect of different potassium concentrations on photosynthetic acclimation of cucumber to elevated CO_2_ concentration. *Soil and Fertilizer Sciences in China* 6, 148–160. doi: 10.11838/sfsc.20180621

Dong, J., Gruda, N., Li, X., Tang, Y., and Duan, Z. (2020). Impacts of elevated CO_2_ on nitrogen uptake of cucumber plants and nitrogen cycling in a greenhouse soil. *Applied Soil Ecology* 145, 103342. doi: 10.1016/j.apsoil.2019.08.004

Dong, J., Li, X., Chu, W., and Duan, Z. (2017). High nitrate supply promotes nitrate assimilation and alleviates photosynthetic acclimation of cucumber plants under elevated CO_2_. *Scientia Horticulturae* 218, 275–283. doi: 10.1016/j.scienta.2016.11.026

Dong, J., Li, X., and Duan, Z. (2016). Biomass allocation and organs growth of cucumber (Cucumis sativusL.) under elevated CO_2_ and different N supply. *Archives of Agronomy and Soil Science* 62, 277–288. doi: 10.1080/03650340.2015.1045497

Dong, J., Li, X., Nazim, G., and Duan, Z. (2018a). Interactive effects of elevated carbon dioxide and nitrogen availability on fruit quality of cucumber (Cucumis sativus L.). *Journal of Integrative Agriculture* 17, 2438–2446. doi: 10.1016/S2095-3119(18)62005-2

Dong, J., Xu, Q., Gruda, N., Chu, W., Li, X., and Duan, Z. (2018b). Elevated and super-elevated CO_2_ differ in their interactive effects with nitrogen availability on fruit yield and quality of cucumber. *Journal of the Science of Food and Agriculture* 98, 4509–4516. doi: 10.1002/jsfa.8976

Dong, Y., Liu, B., Zhang, X., Liu, X., Ai, X., and Li, Q. (2014). Responses of non-structural carbohydrate metabolism of cucumber seedlings to drought stress and doubled CO_2_ concentration. *Chinese Journal of Applied Ecology* 26, 53–60.

Du, X., Song, Y., Pan, L., and Cui, S. (2024). Optimizing Cucumber (Cucumis sativus L.) Fruit Metabolomics Under Elevated CO_2_ and High-Temperature Stress in the Greenhouse. *Horticulturae* 11, 10. doi: 10.3390/horticulturae11010010

Duan J., Cui S., Song Y., Zhang L., Jin L., Pan L., et al. (2023). Influences of high temperature and CO_2_ enrichment on carbohydrate distribution in cucumber seedlings. *Jiangsu Agricultural Sciences* 51, 187–197. doi: 10.15889/j.issn.1002-1302.2023.14.026

Enoch, H. Z., Rylski, I., and Spigelman, M. (1976). CO2_2_ enrichment of strawberry and cucumber plants grown in unheated greenhouses in Israel. *Scientia Horticulturae* 5, 33–41. doi: 10.1016/0304-4238(76)90020-0

Ge, T., and Dabu, X. (2018). Influences of CO_2_ enrichment and different potassium levels on stomatal state of cucumber. *Jiangsu Agricultural Sciences* 46, 110–113. doi: 10.15889/j.issn.1002-1302.2018.17.027

He L., Hai M., and Dabu X. (2017). The Effects of Enriched CO_2_ and K Level on Cucumber Photosynthesis. *Journal of Yunnan Agricultural University (Natural Science)* 32, 57–62. doi: 10.16211/j.issn.1004-390X(n).2017.01.008

He, X., and Li, Q. (2021). Effects of CO_2_ Enrichment and Exogenous ABA on Growth and Contents of Endogenous Hormones of Cucumber Seedlings under Drought Stress. *Journal of Shandong Agricultural University (Natural Science Edition)* 52, 352–357. doi: 10.3969/j.issn.1000-2324.2021.03.002

Hideo, I., and Takaya, O. (1988). Effects of CO_2_ Concentration in the Air, and Shading, on the Utilization of N03 and NH4 by Vegetable Crops. *Journal of the Japanese Society for Horticultural Science* 57, 52–61. doi: 10.2503/jjshs.57.52

Jiang, Y., Cheng, F., Zhou, Y., Xia, X., Shi, K., and Yu, J. (2012). Interactive effects of CO_2_ enrichment and brassinosteroid on CO_2_ assimilation and photosynthetic electron transport in Cucumis sativus. *Environmental and Experimental Botany* 75, 98–106. doi: 10.1016/j.envexpbot.2011.09.002

Kawashiro H., Tsuchiya K., Sakiyama H., and Udagawa Y. (2009). Effects of Low-concentration Carbon Dioxide Supplementation on Fruit Yield and Economic Value of Cucumber on Forced Culture. *Hort. Res. (Japan)* 8, 445–449. doi: 10.2503/hrj.8.445

Kläring, H.-P., Hauschild, C., Heißner, A., and Bar-Yosef, B. (2007). Model-based control of CO_2_ concentration in greenhouses at ambient levels increases cucumber yield. *Agricultural and Forest Meteorology* 143, 208–216. doi: 10.1016/j.agrformet.2006.12.002

Koo, J. K., Hwang, H. S., Hwang, J. H., Park, E. W., Yu, J., Yun, J. H., et al. (2024). Supplemental lighting and CO_2_ enrichment on the growth, fruit quality, and yield of cucumber. *Hortic. Environ. Biotechnol.* doi: 10.1007/s13580-024-00638-y

Larios, B., Agüera, E., De La Haba, P., Pérez-Vicente, R., and Maldonado, J. M. (2001). A short-term exposure of cucumber plants to rising atmospheric CO_2_ increases leaf carbohydrate content and enhances nitrate reductase expression and activity. *Planta* 212, 305–312. doi: 10.1007/s004250000395

Li, D., Dong, J., Gruda, N. S., Li, X., and Duan, Z. (2022a). Elevated root-zone temperature promotes the growth and alleviates the photosynthetic acclimation of cucumber plants exposed to elevated [CO_2_]. *Environmental and Experimental Botany* 194, 104694. doi: 10.1016/j.envexpbot.2021.104694

Li, D., Li, X., Dong, J., Gruda, N. S., and Duan, Z. (2023). Warm root-zone temperature ensures the mineral concentrations in cucumber plants under elevated [CO_2_] by improving the migration pathways of mineral elements from the soil to plants. *Journal of Plant Nutrition and Soil Science* 186, 298–310. doi: 10.1002/jpln.202200361

Li, M., Dong, Y., Cui, Q., Zhang, W., and Ai, X. (2017). Effects of water-nitrogen coupling on the metabolites and key enzyme activities of carbon and nitrogen metabolism in cucumber leaves under doubled CO_2_ concentration. *Plant Physiology Journal* 53, 1717–1727. doi: 10.13592/j.cnki.ppj.2017.0123

Li, M., Li, Y., Zhang, W., Li, S., Gao, Y., Ai, X., et al. (2018a). Metabolomics analysis reveals that elevated atmospheric CO_2_ alleviates drought stress in cucumber seedling leaves. *Analytical Biochemistry* 559, 71–85. doi: 10.1016/j.ab.2018.08.020

Li, Q., Liu, B., and Ai, X. (2010). Effects of doubled CO_2_ concentration on lipid peroxidation and antioxidant system of cucumber seedlings under drought stresses. *Acta Ecologica Sinica* 30, 6063–6071.

Li, Q., Liu, B., Wu, Y., and Zou, Z. (2008). Interactive Effects of Drought Stresses and Elevated CO_2_ Concentration on Photochemistry Efficiency of Cucumber Seedlings. *JIPB* 50, 1307–1317. doi: 10.1111/j.1744-7909.2008.00686x

Li, Q., Liu, B., and Zou, Z. (2011). Effects of Doubled CO_2_ Concentration on Photosynthetic Characteristics of Cucumber Seedlings Under Drought Stresses. *Scientia Agricultura Sinica* 44, 963–971. doi: 10.20103/j.stxb.2010.22.007

Li, S. H., Li, Y. M., Li, M., He, X. R., Jiang, W. L., Liu, B. B., et al. (2022b). CO2 Enrichment Improves the Drought Resistance of Cucumber Seedlings by Improving Antioxidant Capacity and Sucrose Metabolism. *Russ J Plant Physiol* 69, 126. doi: 10.1134/S1021443722060164

Li, S., Li, M., Zhang, W., Li, Y., Ai, X., Liu, B., et al. (2019a). Effects of CO_2_ enrichment on photosynthetic characteristics and reactive oxygen species metabolism in leaves of cucumber seedlings under salt stress. *Acta Ecologica Sinica* 39, 2122–2130. doi: 10.5846/stxb201712212296

Li, S., Li, Y., Gao, Y., He, X., Zhang, D., Liu, B., et al. (2020a). Effects of CO_2_ enrichment on non-structural carbohydrate metabolism in leaves of cucumber seedlings under salt stress. *Scientia Horticulturae* 265, 109275. doi: 10.1016/j.scienta.2020.109275

Li, S., Li, Y., He, X., Li, Q., Liu, B., Ai, X., et al. (2019b). Response of water balance and nitrogen assimilation in cucumber seedlings to CO_2_ enrichment and salt stress. *Plant Physiology and Biochemistry* 139, 256–263. doi: 10.1016/j.plaphy.2019.03.028

Li, X., Chu, W., Dong, J., and Duan, Z. (2014). An Improved High-performance Liquid Chromatographic Method for the Determination of Soluble Sugars in Root Exudates of Greenhouse Cucumber Grown under CO_2_ Enrichment. *J. Amer. Soc. Hort. Sci.* 139, 356–363. doi: 10.21273/JASHS.139.4.356

Li, X., Dong, J., Chu, W., Chen, Y., and Duan, Z. (2018b). The relationship between root exudation properties and root morphological traits of cucumber grown under different nitrogen supplies and atmospheric CO_2_ concentrations. *Plant Soil* 425, 415–432. doi: 10.1007/s11104-017-3555-8

Li, X., Dong, J., Gruda, N., Chu, W., and Duan, Z. (2021). Does the short-term fluctuation of mineral element concentrations in the closed hydroponic experimental facilities affect the mineral concentrations in cucumber plants exposed to elevated CO_2_? *Plant Soil* 465, 125–141. doi: 10.1007/s11104-021-04993-y

Li, X., Dong, J., Gruda, N. S., Chu, W., and Duan, Z. (2020b). Interactive Effects of the CO_2_ Enrichment and Nitrogen Supply on the Biomass Accumulation, Gas Exchange Properties, and Mineral Elements Concentrations in Cucumber Plants at Different Growth Stages. *Agronomy* 10, 139. doi: 10.3390/agronomy10010139

Li, Y., He, X., Li, Q., Liu, B., Li, S., Ai, X., et al. (2019c). Effect of CO_2_ enrichment on antioxidant system in cucumber seedling root system under drought stress. *Plant Physiology Journal* 55, 1011–1019. doi: 10.13592/j.cnki.ppj.2018.0550

Li Y., and Song H. (2023). Effects of CO_2_ Enrichment on Photosynthetic Characteristics and Seedling Quality of Mini-Cucumber in Greenhouse. *Journal of Northeast Agricultural Sciences* 48, 117-120+140. doi: 10.16423/j.cnki.1003-8701.2023.06.024

Li, Y., Song, H., Li, X., Hou, L., and Li, M. (2024). An Analysis of the Mechanism About CO2 Enrichment Promoting Carbohydrate Metabolism in Cucumber (Cucumis sativus L.) Leaves. *International Journal of Molecular Sciences* 25, 11309. doi: 10.3390/ijms252011309

Liu, B. B., Li, M., Li, Q. M., Cui, Q. Q., Zhang, W. D., Ai, X. Z., et al. (2018a). Combined effects of elevated CO_2_ concentration and drought stress on photosynthetic performance and leaf structure of cucumber (Cucumis sativus L.) seedlings. *Photosynt.* 56, 942–952. doi: 10.1007/s11099-017-0753-9

Liu, J., Yan, H., Zhang, Q., and Hou, J. (2016). Plant Growth and Photosynthesis of Grafted Cucumber in Greenhouse Under Sub-High Temperature and Elevated CO_2_ in Short Term Daytime. *Northern Horticulture* 15, 50–54. doi: 10.11937/bfyy.201615013

Liu Y., Sun S., Xing G., Li J., Zhang Z., Yuan H., et al. (2018b). Effects of different concentrations of CO_2_ on the growth and yield of greenhouse cucumber Cucumis sativus L. *Journal of Shanxi Agricultural University (Natural Science Edition)* 38, 53–58. doi: 10.13842/j.cnki.issn1671-8151.201710022

Ma, J., He, C., Yan, Y., Li, Y., and Yu, X. (2012). Effects of Common Soil Cultivation Applying CO_2_ and Organic Soil Cultivation on Yield and Quality of Cucumber in Solar Greenhouse. *China Vegetables* 22, 47–53. doi: 10.19928/j.cnki.1000-6346.2012.22.008

Ma, P., Yang, X., and Li, X. (2003). A Study of Influence of CO_2_ Fertilizer Application on Growth and Development of Cucumber in Greenhouse. *Chinese Journal of Agrometeorology* 09, 49–51. doi: 10.3969/j.issn.1000-6362.2003.04.015

Mortensen, L. M. (1985). Nitrogen Oxides Produced During CO2 enrichment. *New Phytologist* 101, 103–108. doi: 10.1111/j.1469-8137.1985.tb02819.x

Namizaki, H., Iwasaki, Y., and Wang, R. (2022). Effects of Elevated CO2 Levels on the Growth and Yield of Summer-Grown Cucumbers Cultivated under Different Day and Night Temperatures. *Agronomy* 12, 1872. doi: 10.3390/agronomy12081872

Peet, M. M. (1986). Acclimation to High CO2 in Monoecious Cucumbers 1: I. Vegetative and Reproductive Growth. *Plant Physiology* 80, 59–62. doi: 10.1104/pp.80.1.59

Piñero, M. C., Otálora, G., López-Marín, J., and Del Amor, F. M. (2021). Nitrogen management under increased atmospheric CO_2_ concentration in cucumber (Cucumis sativus L.): ameliorating environmental impacts of fertilization. *Sci Rep* 11, 22318. doi: 10.1038/s41598-021-01882-3

Sánchez-Guerrero, M. C., Lorenzo, P., Medrano, E., Baille, A., and Castilla, N. (2009). Effects of EC-based irrigation scheduling and CO_2_ enrichment on water use efficiency of a greenhouse cucumber crop. *Agricultural Water Management* 96, 429–436. doi: 10.1016/j.agwat.2008.09.001

Sánchez-Guerrero, M. C., Lorenzo, P., Medrano, E., Castilla, N., Soriano, T., and Baille, A. (2005). Effect of variable CO_2_ enrichment on greenhouse production in mild winter climates. *Agricultural and Forest Meteorology* 132, 244–252. doi: 10.1016/j.agrformet.2005.07.014

Shibuya, T., Kano, K., Endo, R., and Kitaya, Y. (2018). Effects of the interaction between vapor-pressure deficit and salinity on growth and photosynthesis of Cucumis sativus seedlings under different CO_2_ concentrations. *Photosynt.* 56, 893–900. doi: 10.1007/s11099-017-0746-8

Slack, G., and Hand, D. W. (1985). The effect of winter and summer CO_2_ enrichment on the growth and fruit yield of glasshouse cucumber. *Journal of Horticultural Science* 60, 507–516. doi: 10.1080/14620316.1985.11515658

Slack, G., and Hand, D. W. (1986). The effects of propagation temperature, CO_2_ concentration and early post-harvest night temperature on the fruit yield of January-sown cucumbers. *Journal of Horticultural Science* 61, 303–306. doi: 10.1080/14620316.1986.11515705

Song, H., Li, Y., Xu, X., Zhang, J., Zheng, S., Hou, L., et al. (2020a). Analysis of genes related to chlorophyll metabolism under elevated CO2 in cucumber (Cucumis sativus L.). *Scientia Horticulturae* 261, 108988. doi: 10.1016/j.scienta.2019.108988

Song Y., Du X., Wang P., Lian B., Cui S., and Ye L. (2020b). Effect of CO_2_ Enrichment on Growth and Photosynthetic Characteristic of Cucumber Seedings. *Journal of Inner Mongolia Agricultural University (Natural Science Edition)* 41, 13–19. doi: 10.16853/j.cnki.1009-3575.2020.02.003

Sun, Q., Cui, S., Song, Y., Yang, Z., and Dong, Q. (2016). Impact of Illumination and Temperature Performance of Blanket-inside Solar Greenhouse and CO_2_ Enrichment on Cucumber Growth and Development. *Northern Horticulture* 09, 50–55. doi: 10.16175/j.cnki.1009-4229.2016.08.001

Sun, Q., He, X., Wang, T., Qin, H., Yuan, X., Chen, Y., et al. (2023). The Beneficial Roles of Elevated [CO_2_] on Exogenous ABA-Enhanced Drought Tolerance of Cucumber Seedlings. *Horticulturae* 9, 421. doi: 10.3390/horticulturae9040421

Swedish University of Agricultural Sciences, Alnarp, Sweden, Bergstrand, K.-J., Suthaparan, A., Norwegian University of Life Sciences, Ås, Norway, Mortensen, L. M., Norwegian University of Life Sciences, Ås, Norway, et al. (2016). Photosynthesis in horticultural plants in relation to light quality and CO_2_ concentration. *Europ.J.Hortic.Sci.* 81, 237–242. doi: 10.17660/eJHS.2016/81.5.1

Taub, D. R., Seemann, J. R., and Coleman, J. S. (2000). Growth in elevated CO_2_ protects photosynthesis against high‐temperature damage. *Plant Cell & Environment* 23, 649–656. doi: 10.1046/j.1365-3040.2000.00574x

Tischler, C. R., Polley, H. W., Johnson, H. B., and Pennington, R. E. (2000). Seedling Response to Elevated CO_2_ in Five Epigeal Species. *International Journal of Plant Sciences* 161, 779–783. doi: 10.1086/314304

Wang, X., Hai, M., and Dabu, X. (2013). The Effect of CO_2_ Enrichment and Potassium Supply for the Photosynthate in the Seeding Stage of Cucumber Plants. *Journal of Yunnan Agricultural University (Natural Science)* 28, 676–681. doi: 10.3969/j.issn.1004-390X(n).2013.05.011

Wang, Z., Li, D., Gruda, N. S., Zhu, C., Duan, Z., and Li, X. (2023). How to Efficiently Produce the Selenium-Enriched Cucumber Fruit with High Yield and Qualities via Hydroponic Cultivation? The Balance between Selenium Supply and CO_2_ Fertilization. *Agronomy* 13, 922. doi: 10.3390/agronomy13030922

Yuan, H., Zhou, J., Duan, Z., and Wang, H. (2008). Effects of Elevated CO_2_ Concentration on Growth,Photosynthetic Characteristics and Mineral Elements of Cucumber Seedlings under Salt Stress. *Soils* 05, 797–801. doi: 10.3321/j.issn:0253-9829.2008.05.020

Zhang, L., Song, Y., Zhang, Z., Sun, S., and Pan, L. (2015). Effects of the Starch Accumulation on Photosynthesis of Cucumber Leaves Under Long Term Elevated CO_2_ Condition. *Acta Horticulturae Sinica* 42, 1321–1328. doi: 10.16420/j.issn.0513-353x.2015-0011

Zhang, N., Berman, S. R., van den Berg, T., Chen, Y., Marcelis, L. F. M., and Kaiser, E. (2024). Biochemical versus stomatal acclimation of dynamic photosynthetic gas exchange to elevated CO_2_ in three horticultural species with contrasting stomatal morphology. *Plant, Cell & Environment* 47, 4516–4529. doi: 10.1111/pce.15043
